# Transcriptomic miRNA and mRNA signatures in primary prostate cancer that are associated with lymph‐node invasion

**DOI:** 10.1002/ctm2.70288

**Published:** 2025-04-11

**Authors:** Matias A. Bustos, Kelly K. Chong, Yoko Koh, SooMin Kim, Eleanor Ziarnik, Romela I. Ramos, Gianna Jimenez, David L. Krasne, Warren M. Allen, Timothy G. Wilson, Dave S. B. Hoon

**Affiliations:** ^1^ Department of Translational Molecular Medicine Saint John's Cancer Institute (SJCI) at Providence Saint John's Health Center (SJHC) Santa Monica California USA; ^2^ Department of Urology and Urologic Oncology SJCI at Providence SJHC Santa Monica California USA; ^3^ Department of Genome Sequencing Center SJCI at Providence SJHC Santa Monica California USA; ^4^ Division of Surgical Pathology Providence SJHC Santa Monica California USA

**Keywords:** lymph node dissection, lymph node metastasis, mRNA‐signature, prostate cancer

## Abstract

**Background:**

Nomograms or comparable techniques can be used to determine which patients with prostate cancer (PCa) will benefit from extended pelvic lymph node dissection (ePLND). While nomograms help guide clinical decisions, ∼80% of the patients undergo unnecessary ePLND. This pilot study aims to identify both transcriptomic mRNA and microRNA (miR) signatures in primary PCa tumours that are associated with the presence of lymph node metastasis (LNM).

**Methods:**

Primary PCa tumours obtained from 88 patients (pathologically diagnosed as N0 [pN0, *n* = 44] or as N1 [pN1, *n* = 44]) were profiled using two different probe‐based captured direct assays based on next‐generation sequencing and targeting 19398 mRNA transcripts (human transcriptome panel [HTP] dataset) and 2083 miRs (miRs whole‐transcriptome assay [WTA] dataset). The TCGA‐PRAD (pN0 [*n* = 382] and pN1 [*n* = 70]) and GSE220095 (pN0 [*n* = 138] and pN1 [*n* = 17]) datasets were used for validation using bioinformatic analyses.

**Results:**

A four‐mRNA signature (*CHRNA2*, *NPR3*, *VGLL3* and *PAH*) was found in primary tumour tissue samples from pN1 PCa patients, and then it was validated using the TCGA‐PRAD and GSE220095 datasets. Adding serum prostate‐specific antigen (PSA) values to the four‐gene signature increased the performance to identify pN1 (HTP [AUC = .8487, *p *= 2.18e‐09], TCGA‐PRAD [AUC = .7150, *p *= 8.66e‐08] and GSE220095 datasets [AUC = .8772, *p *= 4.09e‐07]). Paired miR analyses showed that eight miRs were significantly upregulated in primary PCa that were pN1 (*p* < .01). The eight‐miR signature performance increased when adding PSA (WTA dataset [AUC = .8626, *p *= 4.66e‐10]) or Grade group (WTA dataset [AUC = .8689, *p *= 2e‐10]). When combining the miR/mRNA signatures (miR‐663b, *CHRNA2* and *PAH*) with PSA levels, it showed the best performance to distinguish pN1 from pN0 PCa patients.

**Conclusion:**

This study found miR/mRNA signatures in primary PCa tumours that in combination with serum PSA levels may complement nomograms for better detection of PCa patients with LNM and triage patients into better surgical decision‐making.

**Key points:**

- Primary prostate cancer (PCa) tumours from patients pathologically diagnosed as N0 (pN0) or N1 (pN1) were dually assessed for microRNA (miRs) and mRNA levels using an NGS‐based assay.
- A four‐mRNA and an eight‐miRNA signature were found.
- The mRNA signatures were further validated using two datasets.
- The combination of serum prostate‐specific antigen (PSA) levels or Grade Group with the miR/mRNA signatures separates pN1 from pN0 PCa patients.

## Graphical abstract

- Primary prostate cancer (PCa) tumours from patients pathologically diagnosed as N0 (pN0) or N1 (pN1) were dually assessed for microRNA (miRs) and mRNA levels using an NGS‐based assay.
- A four‐mRNA and an eight‐miRNA signatures were found.
- The mRNA signatures were further validated using two datasets.
- The combination of serum prostate‐specific antigen (PSA) levels or Grade Group with the miR/mRNA signatures separate pN1 from pN0 PCa patients.

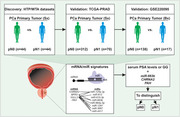

## 1 BACKGROUND

Radical prostatectomy with or without pelvic lymph node dissection (PLND) is one of the main treatment options for early‐stage prostate cancer (PCa) patients, and PLND yields the most accurate and reliable nodal staging.^1^ In clinical practice, PLND is performed for PCa patients with intermediate‐ or high‐risk classification determined by nomograms.^1^, ^2^, ^3^, ^4^, ^5^ However, the positive rate of regional lymph node metastasis (LNM) remains low.^6^, ^7^, ^8^, ^9^ In previous studies, ∼20% of high‐risk patients who received extended PLND (ePLND) had PCa LNM.^1^ Therefore, the majority of PCa patients undergo unnecessary ePLND and have a risk of developing morbid complications such as lymphocele formation, thromboembolic events, vascular injury, nerve injury and ureteral injury.^10^ A major controversy in ePLND is the extent of lymph nodes (LNs) to remove as higher rates of complications are observed when performing super‐ePLND.^10^

Sentinel lymph node biopsy is a procedure established in melanoma and breast cancer that aims to identify metastasis in the LN that directly drains from the primary tumour site intraoperatively, as we have shown.^11^ However, for PCa, the LN drainage is more complex, making the SNLB more challenging.^12^ The utility of radioactive tracers or specific dyes to identify LNM in PCa patients remains questionable due to the discordance amongst procedures and the high false positive rates.^13^, ^14^, ^15^, ^16^, ^17^, ^18^, ^19^, ^20^, ^21^, ^22^ Consequently, more sensitive and specific biomarkers detectable in PCa biopsies before prostatectomy are needed to predict which PCa patients will have LNM and avoid unnecessary PLND.

Germline and somatic mutations are low frequency and limited in primary PCa tumours,^5^, ^23^, ^24^, ^25^, ^26^ while certain genomic alterations, such as *TMPRSS2‐ERG* fusion, are more commonly found.^5^ Therefore, our focus was to identify transcriptomic changes in primary PCa tumours of patients who were histopathology diagnosed with PCa LNM after ePLND. One of the most challenging issues has always been the identification of transcriptome changes in mRNA associated with miR dysregulation using paired analysis on the same tissue section. MiRs are epigenetic regulators of targeted mRNA transcripts and are considered hallmarks of cancer,^27^ and our group showed the importance of miRs in different solid tumours.^26^, ^28^, ^29^, ^30^, ^31^ The combination of paired mRNA and miR analyses on the same tissue section allows for more comprehensive comparisons and better molecular insights into the regulatory networks controlling the cell transcriptome. To our knowledge, there is no report investigating the genome‐wide profiles of mRNA and miR using the same tissue sample and correlating both transcriptomic profiles to clinical and histological parameters of PCa patients.

The present study analysed 88 patients pathologically diagnosed as N0 (pN0) and N1 (pN1) and aimed to (1) define mRNA/miR signatures associated with the presence of LNM in PCa patients, (2) identify the associations between the mRNA/miR signatures and clinicopathology status and (3) explore the immune microenvironment of primary PCa tumours from patients who have LNM. A four‐gene mRNA signature was found in primary PCa tumours from pN1 patients. The four‐gene signature was further validated using the TCGA‐PRAD and GSE220095 datasets.^32^ The mRNA transcriptomic changes were linked to a shorter time to relapse‐free survival (RFS). The downregulation of the four‐gene signature was linked to the upregulation of specific miRs. The upregulated miRs showed an association with shorter RFS. A combinational mRNA/miR signature plus serum prostate‐specific antigen (PSA) levels or grade group (GG) demonstrated better performance to distinguish primary PCa tumours that are pN1 than serum PSA levels or GG alone. Deconvolution analysis showed that M1 macrophages were upregulated in pN1 patients.

## 2 MATERIALS AND METHODS

### 2.1 Study design

Providence SJHC approved this retrospective study under SJHC/SJCI Joint Institutional Review Board (IRB): Universal Consent (Providence Health and Services Portland IRB: JWCI‐18‐0401) and WIRB Copernicus Group, Inc. (WCG) IRB: MORD‐RTPCR‐0995. All participants provided written informed consent. This study enrolled a total of 88 patients who underwent robot‐assisted laparoscopic prostatectomy and ePLND. All patients were pathologically diagnosed with PCa by two experienced surgical pathologists specialised in urology (D.L.K. and W.M.A.). Patients were excluded from the study if they received previous androgen deprivation therapy (ADT), radiation therapy, or any other treatment for PCa. PCa patients with follow‐up periods shorter than one year were excluded. The clinical and pathological information is described in Table 1.

**TABLE 1:** Clinical and pathological variables of prostate cancer (PCa) patients included in the human transcriptome panel (HTP) dataset.

Variables	pN1 group (*n* = 44)	pN0 group (*n* = 44)	*p‐*value
**Age at surgery, median (range)**	65 (52–80)	68 (51–78)	.375
**PSA at surgery (ng/mL), median (range)**	9.10 (3.6–63.4)	6.75 (2.2–32.1)	<.05
**Stage, *n* (%)**			
pT2	8 (18%)	30 (68%)	<.0001
pT3a	15 (34%)	11 (25%)	
pT3b	21 (48%)	3 (7%)	
**GG, *n* (%) **			
GG2	5 (11%)	17 (38.5%)	<.05
GG3	10 (23%)	10 (23%)	
GG4	7 (16%)	4 (9%)	
GG5	22 (50%)	13 (29.5%)	
**BCR**			
No	13 (30%)	34 (77%)	<.0001
Yes	31 (70%)	10 (23%)	

Abbreviations: BCR, biochemical recurrence; GG, grade group; HTG transcriptome panel; pN0, pathologically diagnosed as N0; pN1, pathologically diagnosed as N1; PSA, prostate‐specific antigen.

### 2.2 PCa tissue sample analyses

Formalin‐fixed paraffin‐embedded (FFPE) tissue blocks were cut into 5 µm‐thick sections for hematoxylin and eosin (H&E) staining and microdissection. The microdissected FFPE samples were analysed using the HTG EdgeSeq miRNA whole transcriptome assay (WTA) and HTG EdgeSeq mRNA human transcriptome panel (HTP) assay (HTG). The HTP and WTA assays are extraction‐free and targeted arrays that use histopathologically defined single‐sections of FFPE and Illumina next‐generation sequencing (NGS). The HTP and WTA assays provide a transcriptome panel for 19 398 human mRNA transcripts and 2083 human miR targets, respectively.^33^, ^34^, ^35^ From this point on, we will refer to the data obtained from the HTP or WTA assays as HTP or WTA datasets. For detailed information for sample processing, library generation, data parsing and raw counts, please refer to Supporting information.

### 2.3 Bioinformatics analysis

Data analyses were performed using R version 4.2.1 (R Core Team 2022, https://www.R‐project.org/); ggplot2 (version 3.4.3) was used for data visualisation. The Mann–Whitney U and Fisher's exact tests were used to evaluate differences in pN0 and pN1 patients. Differentially expressed (DE) mRNA and miRs were detected using DESeq2 (version 1.38.3) with a cutoff of a false‐discovery rate (FDR) < .05 and absolute Log_2_ fold change (FC) > |1.5|. For DE analyses were performed with raw counts that were normalised using the package's default median‐of‐ratios method, without further transformation of the data. For DE analysis, only LN positivity was included as a factor (pN0 vs. pN1). Kaplan–Meier survival curves were generated in R using packages survival (3.5‐7) and survminer (0.4.9). For survival analyses the PCa patients were dichotomised into two groups based on the median expression values or the best cut‐off values based on the ROC curve. Correlation analysis of mRNA and miR expression data was conducted in a pairwise manner between a given gene of interest and each miR in the panel, and correlation analysis of mRNA expression data was conducted between each given gene of interest. In both analyses, the Spearman's Rho coefficients and *p*‐values were calculated using the R native cor.test package, and FDR adjustment was conducted by using the p.adjust package. Immune cell fractions were calculated using quanTIseq algorithm.^32^ For the classification of pN status by gene marker expression, receiver operating characteristics (ROC) curve analysis and plots were generated using the R package pROC (1.18.5), while logistic regression model classifiers were fit using the native glm R function. Model *p*‐values were calculated using the log likelihood ratio test in comparison to the null model, while leave‐one‐out cross‐validation accuracy values were calculated using the R package boot (1.3‐28.1). All figures were unified using Adobe Illustrator CC (Adobe) or CorelDraw graphics suite 8X (Corel).

## 3 RESULTS

### 3.1 Patient characteristics and study design

**FIGURE 1:**
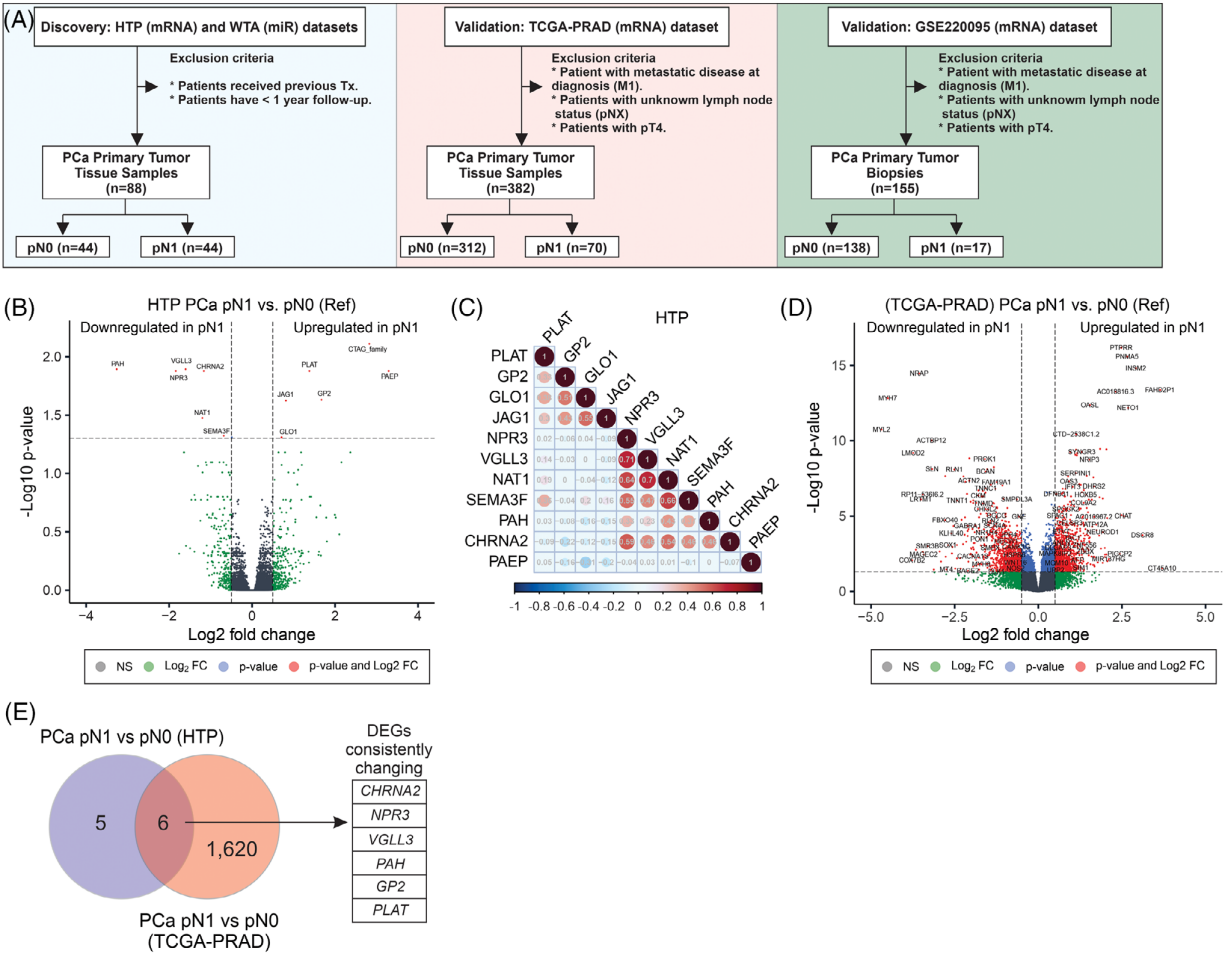
A transcriptomic signature found in primary prostate cancer (PCa) tumours that developed LNM using the human transcriptome panel (HTP) dataset. (A) Schematic representation of the study design. (B) Volcano plot showing the differential expressed genes (DEGs) between primary PCa tumours with pathologically diagnosed as N1 (pN1) (*n* = 44) versus pathologically diagnosed as N0 (pN0) (*n* = 44) status using the HTP dataset. (C) Correlogram showing the correlation values across all the 11 DEGs between pN1 and pN0 groups. (D) Volcano plot showing the DEGs between primary PCa tumours with pN1 (*n* = 70) versus pN0 (*n* = 312) status using the TCGA‐PRAD dataset. (E) Overlapping DEGs in pN1 versus pN0 using the HTP or TCGA‐PRAD datasets. FC, fold change; NS, non‐significant; WTA, whole‐transcriptome assay.

A total of 88 patients diagnosed with primary PCa tumours were included in this retrospective study (Table 1; Figure 1A). There were 44 PCa patients diagnosed with pN1 and 44 patients diagnosed with pN0 status. The median PSA levels at surgery were higher in pN1 than pN0 cases (9.10 ng/mL and 6.75 ng/mL, respectively; *p* < .05). As expected, pN1 patients showed significantly higher pathological T stage and GG, but no significant differences were observed in age at diagnosis (Table 1). The recurrence rate was higher in pN1 as opposed to pN0 cases (70% vs. 23%; *p* < .05). The pN1 group showed a significantly shorter RFS (HR = 4.00; 95% confidence interval, 2.10–7.77, *p* < .0001; Figure S1B).

### 3.2 Transcriptomic patterns found in primary PCa of patients with confirmed LNM

**FIGURE 2:**
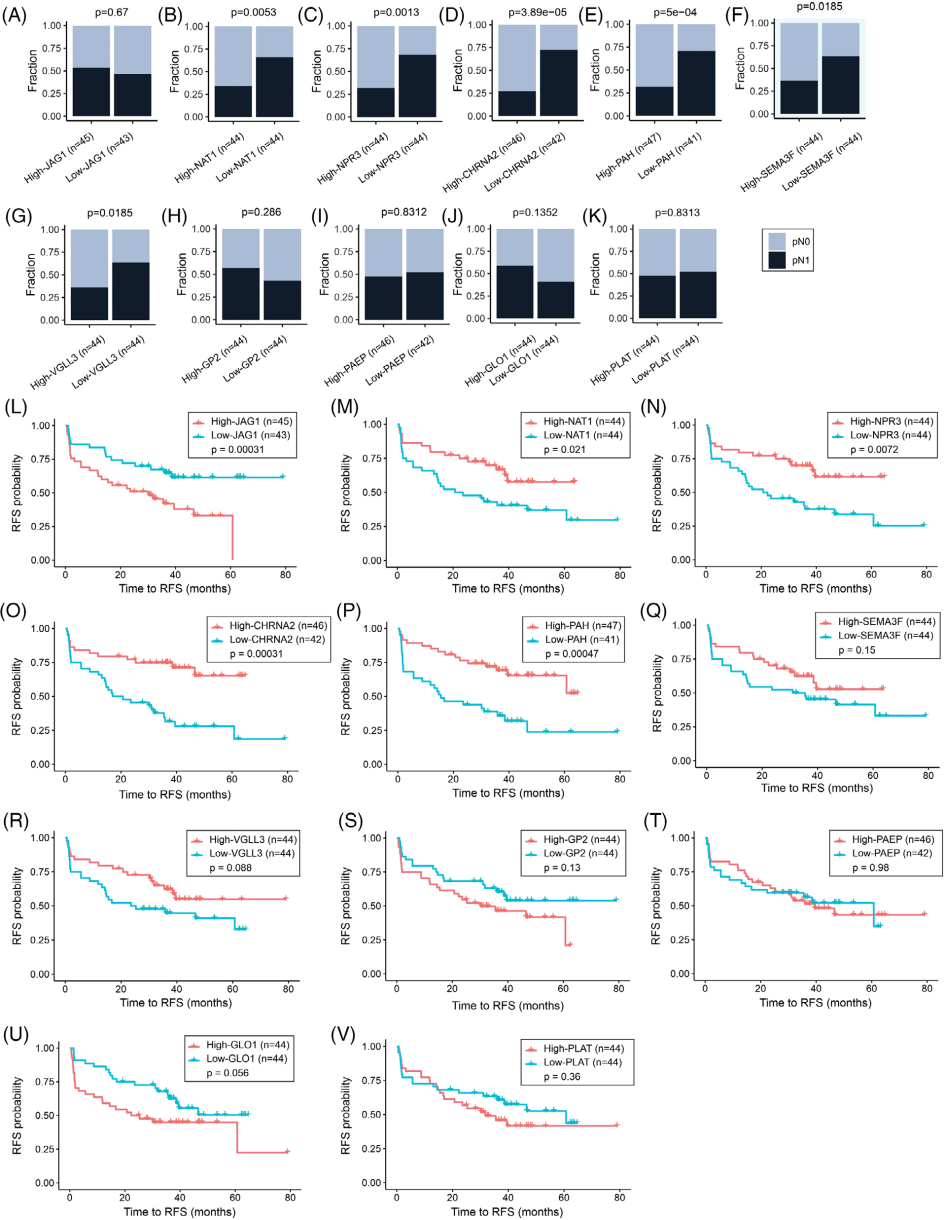
Association between the differentially expressed genes (DEGs) found in pathologically diagnosed as N1 (pN1) and relapse‐free survival (RFS). (A–K) Stacked bar plots showing the distribution of pN1 and pathologically diagnosed as N0 (pN0) patients in low versus high levels considering *JAG1* (A), *NAT1* (B), *NPR3* (C), *CHRNA2* (D), *PAH* (E), *SEMA3F* (F), *VGLL3* (G), *GP2* (H), progestagen associated endometrial protein (*PAEP*) (I), *GLO1* (J), or plasminogen activator tissue type (*PLAT*) (K) genes median expression using the human transcriptome panel (HTP) dataset. (L–V) Kaplan–Meier curves showing the RFS probability comparing patients with low versus high levels considering *JAG1* (L), *NAT1* (M), *NPR3* (N), *CHRNA2* (O), *PAH* (P), *SEMA3F* (Q), *VGLL3* (R), *GP2* (S), *PAEP* (T), *GLO1* (U), or *PLAT* (V) genes median expression using the HTP dataset.

Previous studies demonstrated the utility of HTG EdgeSeq mRNA and miR assays for the molecular profiling of FFPE tumour tissues.^29^, ^30^, ^35^, ^36^, ^37^, ^38^ In this study, primary PCa tumours were obtained from patients pathologically confirmed as pN0 or pN1. Then, primary PCa tumours from both groups were profiled using HTP dataset and compared to identify transcriptomic changes associated with the presence of LNM (Figure 1A and Figure S1A). differentially expressed gene (DEG) analysis comparing pN1 versus pN0 PCa samples showed that six genes were significantly upregulated and six were significantly downregulated. The probe for CTAG_family targets multiple genes, and therefore, the probe was removed from further analysis to avoid confusion (Figure 1B; Table S1). Two different gene clusters were identified using correlation analysis: cluster 1 (*PLAT* [plasminogen activator tissue type], *GP2* [Glycoprotein 2], *GLO1* [Glyoxalase I] and *JAG1* [Jagged canonical notch ligand 1]) and cluster 2 (*NPR3* [Natriuretic Peptide Receptor 3], *VGLL3* [Vestigial like family member 3], *NAT1* [N‐Acetyltransferase 1], *SEMA3F* [Semaphorin 3F], *PAH* [Phenylalanine hydroxylase] and *CHRNA2* [Cholinergic Receptor nicotinic alpha 2 subunit], Figure 1C). To validate the DEGs found in pN1 cases using the HTP dataset, the patients from the TCGA‐PRAD were divided into pN0 (*n* = 312) and pN1 (*n* = 70, Table 2), and both groups were compared to identify DEGs (Figure 1D). We observed that six (*GP2*, *PLAT*, *CHRNA2*, *NPR3*, *VGLL3* and *PAH*) out of the 11 DEGs were consistently changing in the TCGA‐PRAD dataset (Tables S1 and S2). Next, the mRNA levels of the 11 DEGs found in the HTP dataset were divided into high‐ and low‐expression groups based on the median value of mRNA levels for each gene. Then, each group was evaluated for the number of patients with pN1 status. Of note, only low‐*NAT1*, low‐*NPR3*, low‐*CHRNA2*, low‐*PAH*, low‐*SEMA3F*, or low‐*VGLL3* levels were associated with an increased number of pN1 cases (Figure 2A–K; Table 3). Then, each of the 11 DEGs found in the HTP dataset was correlated to RFS data obtained for the 88 PCa patients. Of note, pN1 PCa patients showed a shorter RFS than pN0 (Figure S1B). In Kaplan–Meier analyses, five genes were associated with significant differences in RFS (Figure 2L–V; Table 3). Afterwards, the associations between the expression levels of each gene and the number of patients with recurrent and non‐recurrent PCa were evaluated. Only low‐*NAT1*, low‐*NPR3*, low‐*CHRNA2*, low‐*PAH*, or high‐*JAG1* levels were associated with an increased number of recurrent cases (Figure S2A–K; Table 3). Then, the results from the HTP dataset were validated using the RNA‐Seq data from the TCGA‐PRAD dataset. As expected, patients with pN1 status showed a non‐significant trend to shorter progression‐free survival (PFS, Figure S1D). Notably, low‐*CHRNA2*, low‐*NPR3*, low‐*SEMA3F*, low‐*VGLL3*, high‐*GLO1*, or high‐*JAG1* levels were associated with an increased number of pN1 cases (Figure S3A–K). In the TCGA‐PRAD dataset, low‐*CHRNA2* (*p *= .03), low‐*PAH* (*p *= .029) and high‐*JAG1* (*p *= .049) levels in PCa tissue samples were consistently associated with shorter PFS (Figure S3L–V; Table 2).

**TABLE 2:** Clinical and pathological variables of prostate cancer (PCa) patients of the TCGA‐PRAD dataset.

Variables	pN1 group (*n* = 70)	pN0 group (*n* = 312)	*p‐*value
**Age at surgery, median (range)**	62 (46–75)	62 (41–78)	.75
**PSA at surgery (ng/mL), median (range)**	0.1 (0–37.36)	0.1 (0–39.8)	<.05
**Stage, *n* (%)**			
pT2	2 (3%)	131 (42%)	<.0001
pT3a	17 (24%)	118 (38%)	
pT3b	51 (73%)	63 (20%)	
**GG, *N* (%) **			
GG2	4 (6%)	18 (6%)	<.0001
GG3	9 (13%)	180 (57.5%)	
GG4‐5	57 (81%)	114 (36.5%)	

Abbreviations: GG, grade group; pN0, pathologically diagnosed as N0; pN1, pathologically diagnosed as N1; PSA, prostate‐specific antigen; TCGA‐PRAD, The Cancer Genome Atlas Prostate Adenocarcinoma.

**TABLE 3:** Common differentially expressed genes (DEGs) in tissue samples from pathologically diagnosed as N1 (pN1) versus pathologically diagnosed as N0 (pN0) prostate cancer (PCa) patients using the human transcriptome panel (HTP) or TCGA‐PRAD datasets.

Gene name	DEGs in pN1_vs._pN0 (HTP)	DEGs in pN1_vs._pN0 (TCGA‐PRAD)	Consistent DEGs in HTP and TCGA‐PRAD	DEGs associated with RFS (HTP)	DEGs associated with increased number of pN1 cases (HTP)
***PAH***	Down in pN1	Down in pN1	Yes	Yes	Yes
***NPR3***	Down in pN1	Down in pN1	Yes	Yes	Yes
***CHRNA2***	Down in pN1	Down in pN1	Yes	Yes	Yes
***VGLL3***	Down in pN1	Down in pN1	Yes	No	Yes
***PLAT***	Up in pN1	Up in pN1	Yes	No	No
***GP2***	Up in pN1	Up in pN1	Yes	No	No
***JAG1***	Up in pN1	Up in pN1 (FC 1.48)	No	Yes	No
***GLO1***	Up in pN1	Up in pN1 (FC 1.46)	No	No	No
***SEMA3F***	Down in pN1	Down in pN1 (FC ‐1.22)	No	No	Yes
***PAEP***	Up in pN1	NS	No	No	No
***NAT1***	Down in pN1	NS	No	Yes	Yes

Abbreviations: FC, fold change; HTG transcriptome panel; NS, non‐significant; RFS, relapse‐free survival; TCGA‐PRAD, The Cancer Genome Atlas Prostate Adenocarcinoma.

The mRNA expression levels of the 11‐gene signature were analysed in the 33 tumour types of the TCGA database to assess specificity of the PCa mRNA signature. Of note, primary PCa tumours showed high *JAG1*, *GP2*, *GLO1*, *NPR3*, *VGLL3*, *CHRNA2* and *SEMA3F* mRNA levels in primary PCa tissue samples (Figure S4–5). In addition, *CHRNA2* and *GP2* showed a PCa tissue‐specific expression (Figure S4–5). Overall, these results suggest that specific transcriptomic changes found in primary PCa tumours of pN1 cases may be associated with more aggressive primary PCa tumours with higher rates of recurrence, while the downregulation of *PAH*, *NPR3* and *CHRNA2* mRNA levels may be more implicated in the metastatic development of PCa LNM.

### 3.3 Validation of the mRNA signature in PCa primary tumours

**FIGURE 3:**
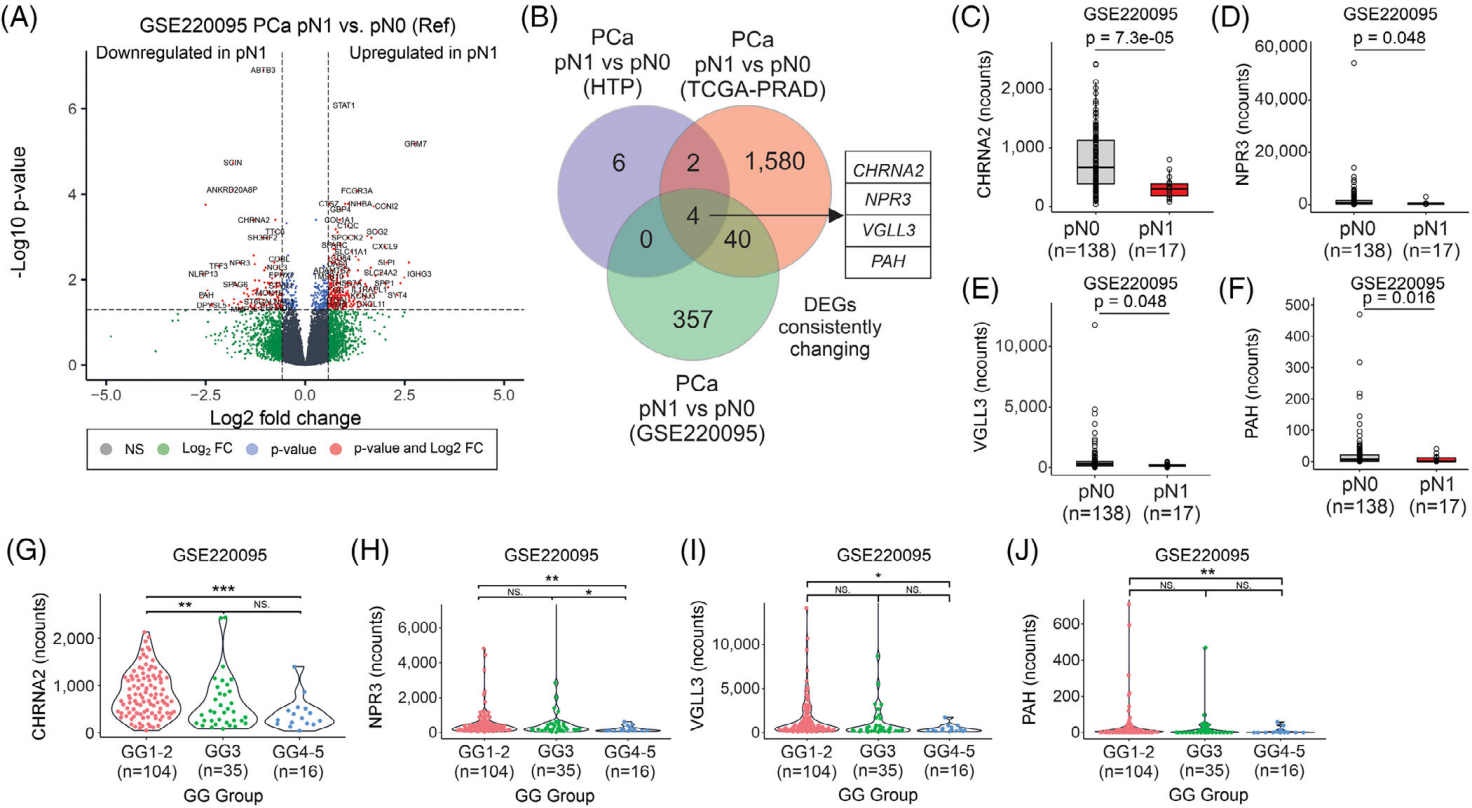
A four‐mRNA signature found in prostate cancer (PCa) tissue biopsies. (A) Volcano plot showing the differential expressed genes (DEGs) between primary PCa tumours with pathologically diagnosed as N1 (pN1) (*n* = 17) versus pathologically diagnosed as N0 (pN0) (*n* = 138) status in the GSE220095 dataset. (B) Overlapping DEGs in pN1 versus pN0 across all the comparisons. (C–F) Boxplots showing the mRNA levels of *CHRNA2* (C), *NPR3* (D), *VGLL3* (E) and *PAH* (F) in pN0 versus pN1 groups in the GSE220095 dataset. (G–J) Violin plots showing the mRNA levels for *CHRNA2* (G), *NPR3* (H), *VGLL3* (I) and *PAH* (J) in grade group (GG)1‐2, GG3 and GG4‐5 in the GSE220095 dataset. FC, fold change; HTP, human transcriptome panel; NS, non‐significant.

The observations in the HTP and TCGA‐PRAD datasets were further validated using the RNA‐Seq data from GSE220095 dataset,^39^ which contains FFPE tissue biopsies from primary tumours obtained from 155 PCa patients with clinical annotations (Figure 1A). PCa patients were divided into pN0 (*n* = 138) and pN1 (*n* = 17) groups (Table 4). As expected, pN1 PCa patients showed a significantly shorter time to biochemical recurrence (BCR, Figure S1F). The pN1 group showed 401 DEGs compared to the pN0 group (Figure 3A; Table S3). Four overlapping genes (*CHRNA2*, *NPR3*, *VGLL3* and *PAH*) were consistently and significantly downregulated in pN1 groups in the three comparisons (Figure 3B–F, Figure S6A–H, and Table S4). Then, the mRNA levels of the four genes (*CHRNA2*, *NPR3*, *VGLL3* and *PAH*) in the three datasets were analysed across GG. Notably, the four genes were significantly downregulated in the GG4‐5 group compared to the GG1‐2 group (Figure 3G–J and Figure S6I–P). In a separate analysis the four genes found in pN1 were categorised based on median mRNA levels into low and high groups. Of note, PCa patients with low mRNA levels for *CHRNA2*, *NPR3* and *VGLL3* showed significantly shorter time to BCR in the GSE220095 dataset (Figure S7A–D). Also, PCa patients with low levels of *CHRNA*2, *NPR3* and *VGLL*3 showed a significantly increased number of cases with pN1 status (Figure S7E–H). These results showed that *CHRNA2*, *NPR3*, *VGLL3* and *PAH* are downregulated in tissue biopsies taken from primary PCa tumours of pN1 patients, suggesting that the four‐mRNA signature may have potential in the identification of pN1 PCa patients eligible for ePLND.

**TABLE 4:** Clinical and pathological variables of the prostate cancer (PCa) patients obtained from the GSE220095 dataset.

Variables	pN1 group (*n* = 17)	pN0 group (*n* = 138)	*p‐*value
**Age at surgery, median (range)**	67 (51–79)	67 (42–79)	.475
**PSA at surgery (ng/mL), median (range)**	18 (4.3–68.58)	7.8 (1–76.79)	<.001
**Stage, *n* (%)**			
pT2	2 (12%)	107 (77.5%)	<.0001
pT3a	3 (18%)	22 (16%)	
pT3b	12 (70%)	9 (6.5%)	
**GG, *n* (%) **			
GG2	4 (24%)	100 (72.5%)	<.0001
GG3	7 (41%)	28 (20%)	
GG4‐5	6 (35%)	10 (7.5%)	

Abbreviations: GG, grade group; pN0, pathologically diagnosed as N0; pN1, pathologically diagnosed as N1; PSA, prostate‐specific antigen.

### 3.4 A four‐gene mRNA signature distinguishes pN1 from pN0 PCa patients

**FIGURE 4:**
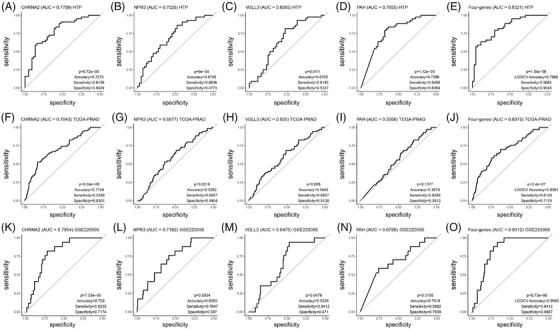
Receiving operating characteristics (ROC) curves for the four‐mRNA signature. (A–E) ROC curves showing the performance of *CHRNA2* (A), *NPR3* (B), *VGLL3* (C), *PAH* (D) and four genes (E) to distinguish pathologically diagnosed as N1 (pN1) from pathologically diagnosed as N0 (pN0) groups in the human transcriptome panel (HTP) dataset. (F–J) ROC curves showing the performance of *CHRNA2* (F), *NPR3* (G), *VGLL3* (H), *PAH* (I) and four genes (J) to distinguish pN1 from pN0 groups in the TCGA‐PRAD dataset. (K–O) ROC curves the performance of *CHRNA2* (M), *NPR3* (N), *VGLL3* (O), *PAH* (P) and four genes (Q) to distinguish pN1 from pN0 groups in the GSE220095 dataset.

To demonstrate the clinical utility, we determined the performance of each of the four genes in the signature using ROC curves. Overall, lower AUC values were observed in the TCGA‐PRAD dataset compared to the HTP or GSE220095 datasets (Figure 4A–O). The highest AUC values were observed for *CHRNA2* in the three datasets analysed (Figure 4A–O; Table S5). The four‐gene signature showed good performance in the HTP, the TCGA‐PRAD, as well as in the GSE220095 datasets (Figure 4E,J,O; Table S5), suggesting a potential value to distinguish patients with primary PCa tumours who have LNM. When PCa patients were divided based on ROC cutoff values (Table S6), instead of the median cutoff values, the associations between miRs and RFS or pN status significantly improved for most of the comparisons (Figure S8).

### 3.5 Overlapping mRNA signatures in aggressive primary PCa tumours

In this section, we focused on the identification of specific transcriptomic signatures in primary tumours from PCa patients that were recurrent versus non‐recurrent. As expected, patients with recurrent PCa showed worse outcomes in the HTP, TCGA‐PRAD and GSE220095 datasets (Figure S1C,E,G). Several DEGs were observed in each comparison (Figure S9A–C; Table S7–9); however, only non‐SMC condensin II complex subunit D3 (*NCAPD3*) was commonly found across the three datasets (Figure S10D). Although this is not the focus of this study, functional assays showed that *NCAPD3* is activated by the AR signalling and *NCAPD3* upregulation promotes PCa progression.^40^, ^41^, ^42^ Of note, *NPR3*, *VGLL3* and *PLAT* were significantly changing in the HTP and GSE220095 datasets, but not in the TCGA‐PRAD dataset (Figure S10D). To summarise, elevated mRNA levels of *NCAPD3* may be associated with aggressive primary PCa tumours.

### 3.6 The miR and mRNA levels were negatively correlated in primary PCa tumours of patients who develop LNM

**FIGURE 5:**
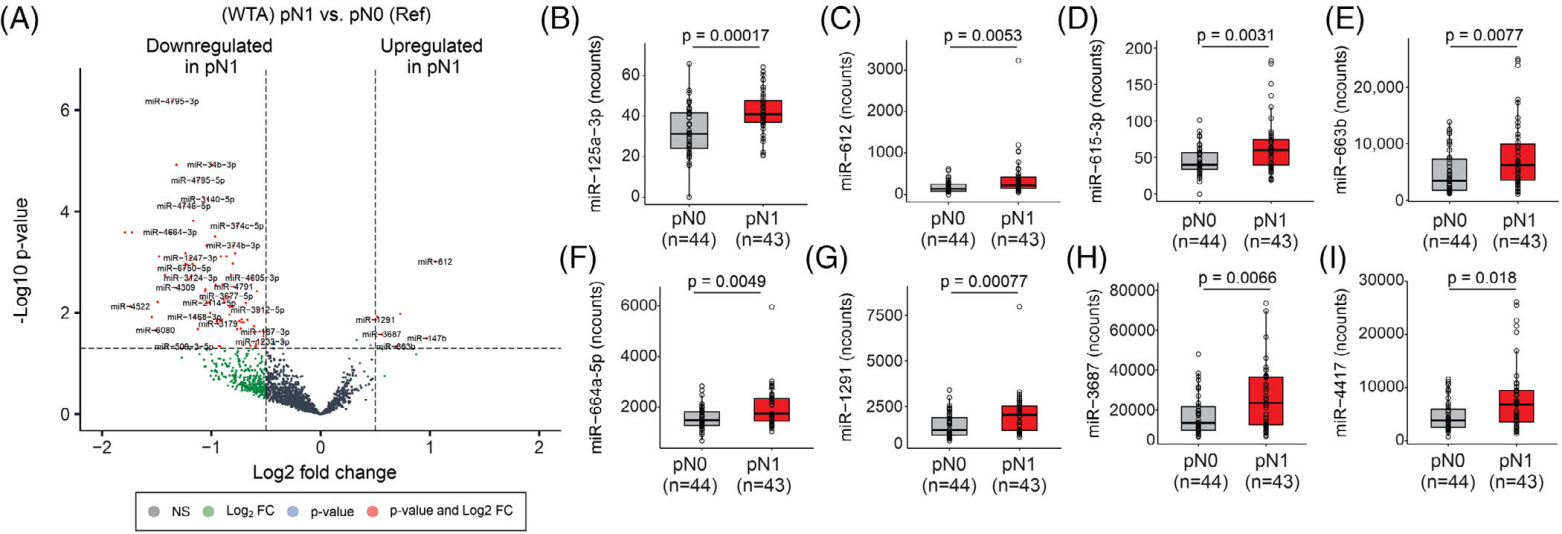
Upregulated miRs in primary prostate cancer (PCa) tumours that developed LNI. (A) Volcano plot showing the DE miRs between primary PCa tumours with pathologically diagnosed as N1 (pN1) (*n* = 44) versus pathologically diagnosed as N0 (pN0) (*n* = 44) status in the whole‐transcriptome assay (WTA) dataset. (B–I) Box plots showing the levels of miR‐125a‐3p (B), miR‐612 (C), miR‐615‐3p (D), miR‐663b (E), miR‐664a‐5p (F), miR‐1291 (G), miR‐3687 (H) and miR‐4417 (I) between pN1 versus pN0 groups. FC, fold change; NS, non‐significant.

**FIGURE 6:**
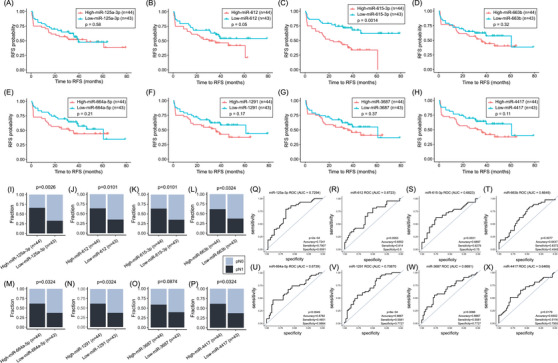
Upregulated miRs found in primary prostate cancer (PCa) tumours that developed LNI were associated with relapse‐free survival (RFS) and pN status. (A–H) Kaplan–Meier curves showing the RFS probability comparing patients with low versus high levels of miR‐125a‐3p (A), miR‐612 (B), miR‐615‐3p (**c**), miR‐663b (D), miR‐664a‐5p (E), miR‐1291 (F), miR‐3687 (G) and miR‐4417 (H) in the whole‐transcriptome assay (WTA) dataset. (I–P) Stacked bar plots showing the distribution of pathologically diagnosed as N1 (pN1) and pathologically diagnosed as N0 (pN0) patients with low versus high levels of miR‐125a‐3p (I), miR‐612 (J), miR‐615‐3p (K), miR‐663b (L), miR‐664a‐5p (M), miR‐1291 (N), miR‐3687 (O) and miR‐4417 (P) in the WTA dataset. (Q–X) Receiving operating characteristics (ROC) curves the performance of miR‐125a‐3p (Q), miR‐612 (R), miR‐615‐3p (S), miR‐663b (T), miR‐664a‐5p (U), miR‐1291 (V), miR‐3687 (W) and miR‐4417 (X) to distinguish pN1 from pN0 groups in the WTA dataset.

In this study, the WTA (miR) and HTP (mRNA) datasets provided paired profiles for each PCa tissue sample. Initially, the comparison between the miR profiles of primary PCa tumours from pN1 versus pN0 patients showed 103 miRs DE (Figure 5A, Figure S1H, and Table S10), of which only ten were upregulated in pN1 primary PCa tumours. Then, the profiles of the DE miRs were correlated with the 11 DEGs found between pN1 versus pN0. Only the DE miRs in pN1 group that significantly correlated (> .25 or <−.25) with any of the 11 DEGs in pN1 were included (Table S11). Ten miRs (miR‐3188, miR‐4632‐3p, miR‐6729‐3p, miR‐4745‐3p, miR‐4791, miR‐5582‐5p, miR‐323a‐5p, miR‐6799‐3p, miR‐4695‐3p and miR‐548h‐5p) were significantly downregulated in pN1 and negatively correlated with the five upregulated genes found in pN1 primary PCa (Table S12). On contrary, eight miRs (miR‐125a‐3p, miR‐612, miR‐615‐3p, miR‐663b, miR‐664a‐5p, miR‐1291, miR‐3687 and miR‐4417) were significantly upregulated in pN1 and negatively correlated with the six downregulated genes found in pN1 (Figure 5B–I; Table 3, and Table S12). The eight upregulated miRs found in pN1 primary PCa tumours were stratified based on the miR detection levels into high and low groups and related to RFS or pN status. Only miR‐612 and miR‐615‐3p were significantly associated with a shorter RFS (Figure 6A–H; Table 5). The number of pN1 cases significantly increased in PCa patients with high levels of any of the miRs found, except for miR‐3687 (Figure 6I–P; Table 5). Each of the eight miRs showed good performance to distinguish pN1 from pN0 PCa patients in the WTA dataset (Figure 6Q–X; Table S5). Using ROC cutoff values (Table S6) improved the associations observed between miRs and RFS or pN status (Figure S10).

**TABLE 5:** Upregulated miRs in primary tumour tissues from pathologically diagnosed as N1 (pN1) versus pathologically diagnosed as N0 (pN0) patients using the whole‐transcriptome assay (WTA) dataset.

Probe name	pN1 vs. pN0 (WTA)	Upregulated miRs associated with RFS (WTA)	Upregulated miRs associated with increased number of pN1 cases (WTA)	Upregulated miRs associated with RFS and increased number of pN1 cases (WTA)
miR‐125a‐3p	Up in pN1	No	Yes	No
miR‐612	Up in pN1	Yes	Yes	Yes
miR‐615‐3p	Up in pN1	Yes	Yes	Yes
miR‐663b	Up in pN1	No	Yes	No
miR‐664a‐5p	Up in pN1	No	Yes	No
miR‐1291	Up in pN1	No	Yes	No
miR‐3687	Up in pN1	No	Yes	No
miR‐4417	Up in pN1	No	Yes	No

Abbreviations: miR, microRNA; PCa, prostate cancer; RFS, relapse‐free survival; TCGA‐PRAD, The Cancer Genome Atlas Prostate Adenocarcinoma.

Specific miRs (such as miR‐125a‐3p) showed negative correlations with multiple genes *NPR3, CHRNA2, NAT1*, *VGLL3*, and *SEMA3F*, suggesting a common regulatory mechanism (Table S12). Conversely, four genes (*NPR3*, *NAT1*, *VGLL3*, and *SEMA3F*) were negatively correlated with more than one miR (miR‐125a‐3p, miR‐663b, miR‐4417 and miR‐3687, Table S12). These results suggest that upregulated or downregulated miRs in pN1 cases may promote LNM by modifying the gene expression output of primary PCa tumours. Therefore, we assessed the TargetScan database to determine predictive miRs targeting any of the 11 mRNA previously identified. We observed that *NPR3*, which is downregulated in pN1, was a predictive target for miR‐125a‐3p, miR‐663b and miR‐664a‐5p (Table S13). Of notice, miR‐125a‐3p, miR‐663b and miR‐664a‐5p were upregulated in pN1 PCa tumours and showed a negative correlation with *NPR3* (Figure 6I–P; Table S13). These results suggest that miRs may promote the downregulation of *CHRNA2, NPR3, VGLL3* and *PAH* genes, which are associated with primary PCa tumours that developed LNM.

### 3.7 Combination of miR/mRNA signatures and PSA or GG levels distinguished primary PCa tumours that developed LNM

**FIGURE 7:**
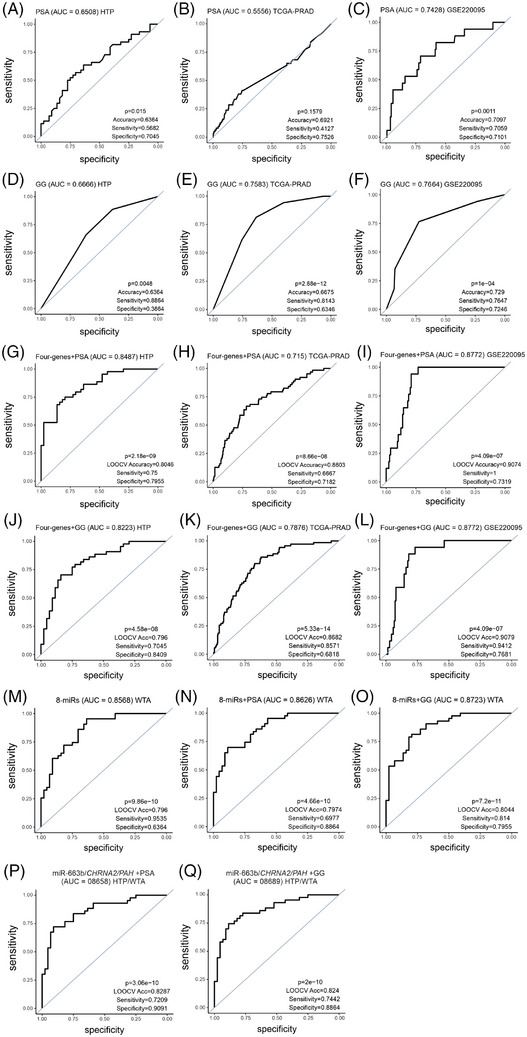
Receiving operating characteristics (ROC) curves for combinational miR/mRNA signature found in primary prostate cancer (PCa) tumours that developed LNI. (A–Q) ROC curves showing the performance of: serum prostate‐specific antigen (PSA) levels to distinguish pathologically diagnosed as N1 (pN1) from pathologically diagnosed as N0 (pN0) groups in the human transcriptome panel (HTP) dataset (A), in the TCGA‐PRAD dataset (B), or in the GSE220095 dataset (C); grade group (GG) to distinguish pN1 from pN0 groups in the HTP dataset (D), in the TCGA‐PRAD dataset (E), or in the GSE220095 dataset (F); four‐genes signature plus serum PSA levels to distinguish pN1 from pN0 groups in the HTP dataset (G), in the TCGA‐PRAD dataset (H), or in the GSE220095 dataset (I); four‐genes signature plus GG to distinguish pN1 from pN0 groups in the HTP dataset (J), in the TCGA‐PRAD dataset (K), or in the GSE220095 dataset (L); the eight‐miRs signature (M), the eight‐miRs signature plus serum PSA levels (N) and the eight‐miRs signature plus GG (O) in the whole transcriptome panel (WTA) dataset; miR‐663b, *CHRNA2*, *PAH*, plus serum PSA levels (P), or miR‐663b, *CHRNA2*, *PAH*, plus GG (Q) to distinguish pN1 from pN0 groups in the HTP and WTA datasets.

Then, the miR and mRNA signatures obtained from the HTP and WTA datasets were combined with the serum PSA levels or GG. Adding the serum PSA levels or GG to the four‐gene signature improved the performance of the four‐gene signature alone, the serum PSA levels alone, or GG alone in the three datasets (Figure 7A–L; Table S5). Then, we also compared the performance of the eight‐miRs as a signature or the eight‐miRs combined with serum PSA levels or GG. The eight‐miRs signature demonstrated good performance, and the combination with the serum PSA levels or GG slightly improved the performance in the HTP dataset (Figure 7M–O; Table S5). Finally, we weighed the miR and mRNA using log likelihood *p*‐value. The combination of miR‐663b, *CHRNA2*, *PAH* and serum PSA levels or GG showed a good performance in the HTP dataset (Figure 7P–Q; Table S5). To summarise, this study identified unique miR and mRNA, as well as miR/mRNA signatures that combined with serum PSA levels or GG significantly improved the detection of primary PCa tumours that developed LNM.

### 3.8 Immune cells of primary PCa tumour microenvironment are associated with pN1 status or RFS

Previous studies have shown that PCa tumours are immune ‘cold’, and therefore, immunotherapies showed no advantage for the treatment of patients with primary PCa.^43^ Cancer‐associated fibroblast, macrophages, natural killer (NK) cells, mast cells and T cells are commonly found infiltrated in primary PCa tumours of patients and are associated with survival and metastasis.^44^ Thus, we focused on understanding the types and fractions of the immune cells in primary PCa and their relation to clinical outcomes. Initially, we evaluated whether IFN‐γ score^45^ was a factor associated with recurrence, pN status, or RFS in PCa patients. However, the IFN‐γ score showed no association with any of the variables analysed (Figure S11A–C). CD8^+^ and CD3^+^ T cell populations are associated with survival in different solid tumours.^32^ Of note, neither CD8^+^ nor CD3^+^ T cell populations were associated with pN status, recurrence, or RFS in the HTP dataset (Figure S12A–E). Then, we applied the quanTIseq algorithm^32^ to the HTP dataset to obtain immune cell fraction estimates (Figure S13A–B). Of the ten immune cell types analysed, only macrophages M1 were significantly upregulated in pN1 cases (*p* = .01, Figure S14A–K). Interestingly, low‐macrophage M2 macrophages or low‐NK cell fractions were significantly correlated with shorter time for RFS (Figure S13C–F). These results suggest that immune cell types such as M2‐macrophages or NK cells are associated with shorter RFS. Only M1‐macrophages showed increased levels in primary PCa tumours that developed LNM.

## 4 DISCUSSION

A current long‐term unmet problem is to determine which PCa patients should receive prostatectomy and ePLND. The decision on performing ePLND depends on current nomograms.^9^ The nomograms have low specificity, and consequently ∼80% of PCa patients undergo unnecessary ePLND, which increases the risk of postsurgical morbidity and complications.^10^ A recent study by Di Pierro et al. showed that most currently used nomograms have similar performance, even in high‐risk PCa patients.^9^ On the other hand, ePLND helps with accurate staging and prognosis of PCa patients, which is a limitation of current imaging technologies.^9^, ^10^ The presence of LNI changes the treatment management from local and curative surgery to adjuvant ADT with or without radiotherapy.^9^, ^10^ Therefore, there is a need to optimise the current nomograms to better select high‐risk and intermediate‐risk patients eligible for ePLND.

Previous studies in solid tumours demonstrated the reproducibility between the HTP and bulk RNA‐seq assays, as well as the utility of the HTP assay.^46^, ^47^ The HTP assay is an extraction‐free method that relies on the NGS‐based quantification. In this study, the mRNA and miR levels were profiled on a single tissue section by using the same tissue lysate for mRNA (using HTP) and miR (using WTA) analyses. The dual assessment of both mRNA and miR molecules using a single FFPE section represents a concurrent direct assay that may have potential immediate application to molecular biomarker development, especially in small tissue specimens, such as tissue biopsies. Using this approach, we develop a four‐gene signature (*CHRNA2*, *NPR3*, *VGLL3* and *PAH*) able to stratify PCa patients who have LNM, with better performance than serum PSA levels or GG alone. Little is known about the function of *CHRNA2*, *NPR3*, *VGLL3* and *PAH* genes in PCa. *CHRNA2* encodes an acetylcholinergic receptor subunit and is located on the 8p21 locus.^48^ PCa patients treated with ADT showed significantly higher *CHRNA2* mRNA levels.^49^ In addition, CHRNA2 is a downstream target of the androgen receptor signalling pathway and is shown to be repressed in vitro by DHT treatment.^50^ Fan et al. identified a 13‐gene signature that included *CHRNA2* based on TCGA‐PRAD, MSKCC and GEO databases;^49^ however, in that study, *CHRNA2* did not correlate with RFS. In a recent study, Pudova et al. identified seven genes (*CST2*, *F5*, *OCLN*, *PCAT4*, *RAB27A*, *TBX1* and *VGLL3*) in pN1 primary PCa tumours. As part of the four‐gene signature identified, *VGLL3* was found consistently downregulated in the HTP, TCGA‐PRAD and GSE220095 datasets. However, the results obtained from both independent cohorts by Pudova et al. showed downregulation of *VGLL3* in frozen samples and upregulation of *VGLL3* in FFPE tissue samples.^51^

In the last years, there have been tremendous efforts in the identification of gene signatures associated with clinical outcomes in early‐stage PCa patients.^39^ In this study, the authors analysed PCa tissue biopsies and identified a 204‐genes signature with better predictive values than clinical and histological parameters. However, there are more limited studies with a focus on transcriptomic signatures that allow for the identification of primary tumours that develop LNM. Previous studies have tried to identify transcriptomic signatures in primary PCa tumours associated with the development of LNM.^24^, ^51^ For example, the transcriptomic signatures identified by qRT‐PCR combined with pathological stage (*CST2*+*OCLN*+pT) showed variable predictive values (AUC ranges from .84 to 1.00) for LNM using different algorithms.^51^ A limitation of previous analysis is the lack of correlation between the transcriptomic signatures and clinical outcomes. In this study, three of the genes in the four‐gene signature showed a prognostic utility for PCa patients in the HTP and GSE220095 datasets, although there are some differences in clinical endpoints across the datasets (e.g., RFS and BCR).

MiRs are a hallmark of cancer, and >2000 miRNAs have been identified in the human genome.^52^, ^53^ MiRs regulate gene expression output by post‐transcriptionally degrading a defined set of mRNA targets.^52^, ^53^ The regulation of mRNA by miR is complex, and the relation between miR and mRNA is not always one‐to‐one.^52^, ^53^ For example, a single miR can regulate hundreds of mRNA, and conversely, a single mRNA can be targeted by several miRs.^54^ The miR‐mRNA analyses are relevant to understanding how both types of molecules integrate in the complex regulatory networks that govern the transcriptional fate of tumour cells.^52^, ^53^ These regulatory networks define the biological process and the signalling pathways responsible for cancer initiation, progression and metastatic cascade.^52^, ^53^ In this study, 2083 miRs were profiled, and miR signatures were correlated with pN1 status, RFS and clinical outcomes.

Only miR‐615‐3p found in this study overlapped with the miR signature previously proposed by Pudova et al.^55^ Of note, the differences in the miRs found may arise from the analytical methodology (PCR‐based vs. NGS‐based) as well as the extraction processes utilised. Also, Pudova et al. demonstrated that miR‐615‐3p is upregulated in primary tumours using two datasets and showed that tissue samples from LNM showed significantly higher levels of miR‐615‐3p than PCa primary tumours.^51^, ^56^ Supporting our findings, a previous study by Laursen et al. demonstrated that miR‐615‐3p is an independent predictive factor for postoperative BCR and PCa‐specific survival.^57^ Functionally, miR‐615‐3p overexpression correlates with enhanced proliferation and migration of PCa cell lines.^57^ Also, miR‐125a was significantly upregulated in tissue samples obtained from high‐risk PCa patients.^58^

It is worth noting that ∼90% of the DE miRs were downregulated in primary PCa tumours of pN1 versus pN0 groups. However, our focus was on the identification of miR signatures that could explain the downregulation of the four mRNA genes consistently found. We found that the four‐gene signature was linked to a potential regulatory miR‐driven mechanism, using predicting tools for miR targets.

The addition of serum PSA levels or GG to the eight‐miR signature slightly improved the performance. Comparable results were observed when adding the serum PSA levels or GG to the four‐gene signature. Additionally, a miR/mRNA signature in combination with serum PSA levels improved the performance observed for the four‐signature alone in the three datasets or in combination with serum PSA levels in two out of three datasets. Therefore, the results of miR/mRNA signature may offer new alternative biomarkers that, in combination with serum PSA levels, may improve the detection of primary PCa tumours developing LNM.

Primary prostate tumours are inherently immune “cold” tumours and show: (1) low tumour mutation burden and low number of new neoantigens, (2) decreased or low levels of MHC class I and II in antigen‐presenting cells, (3) increased levels of myeloid‐derived suppressor cells, regulatory T cells, tumour‐associated macrophages and cancer‐associated fibroblasts and (4) an immunosuppressive cytokine profile enriched in TGF‐β.^12^ There is conflicting information available about tumour‐infiltrating lymphocytes (TILs) and clinical outcomes.^59^, ^60^, ^61^, ^62^ In the HTP dataset, we observed a larger fraction of neutrophils, NK cells and M2‐macrophages compared to other immune cells. Consistently with recent studies,^63^, ^64^ low‐NK cells or low M2‐macrophages fractions were associated with shorter RFS in PCa. When investigating the immune cell populations enriched in primary PCa tumours pN1, we found significantly higher levels of M1 macrophages. Conversely, Ntala et al. observed a significant, but borderline, decrease M1‐macrophages tumour infiltration in primary PCa tumours.^65^ The differences in the results may arise from the variations in the techniques utilised and the cohorts of patients under study. While Ntala et al. assessed CD68 and CD163 markers by multiplex immunofluorescence, we used quanTIseq algorithm that utilised several curated gene‐signatures to identify ten immune cell populations.^32^ Overall, these results suggest that specific immune cell populations may play a significant role in primary tumour from pN1 PCa patients; however, more experimental evidence is needed to further validate these findings.

## 5 CONCLUSION

This study determined transcriptomic changes in primary PCa tumours using extraction‐free NGS‐based technology and found gene signatures that are associated with PCa patients who have LNM. In paired‐tissue sample analysis, the identification of regulatory miRs targeting genes of the four‐gene signature suggested a potential link between miR dysregulation and the transcriptomic changes associated with PCa patients who have LNM. The addition of PSA levels or GG significantly increased the performance of the four‐gene signature or a combinational miR‐mRNA signature (miR‐663b/*CHRNA2*/*PAH*). The validation of the four‐gene signature in core biopsies suggested the potential application of the signature to the stratification of PCa patients eligible for ePLND before prostatectomy. A prospective study and larger cohorts of tissue biopsies from PCa patients are needed to demonstrate the clinical utility of the miR/mRNA signatures alone or in combination with current nomograms in detecting PCa patients who should undergo ePLND.

## AUTHOR CONTRIBUTIONS

Matias A. Bustos designed the study, performed analysis, organized and designed the figures, project management, and wrote the original manuscript. Kelly K. Chong performed bioinformatic and statistical analyses and wrote the original manuscript. Yoko Koh collected and prepared samples, conducted HTP and WTA assays, tissue pre‐processing and microdissection, and wrote the original manuscript. SooMin Kim and Eleanor Ziarnik performed mRNA (HTP) and miR (WTA) library processing and sequencing. Romela I. Ramos project organization. Gianna Jimenez performed clinical data organization. David L. Krasne and Warren M. Allen performed pathological FFPE tissue samples review and data curation. Timothy G. Wilson clinical samples, clinical data organization, project management, clinical data review, and funding acquisition. Dave S. B. Hoon project organization and management. All authors reviewed, edited, and approved the final version of the manuscript.

## CONFLICT OF INTEREST STATEMENT

The authors declare no conflicts of interests.

## ETHICS STATEMENT

The study was conducted following the Declaration of Helsinki. Human samples and clinical information for this study were obtained according to the protocol guidelines approved by the SJHC/SJCI Joint Institutional Review Board (IRB) and WIRB Copernicus Group, Inc. (WCG) IRB: MORD‐RTPCR‐0995. All participants provided written informed consent.

## Supporting information



Supporting information

Supporting information

## Data Availability

The data presented is publicly available in the GEO database under GSE220095.^39^ The TCGA‐PRAD data is publicly available at https://xenabrowser.net/. The transcriptome mRNA and miR data generated using the HTP and WTA assays is provided within the supporting information files (Table S14‐16).
