# Supplementary material for: Transcriptomic miRNA and mRNA signatures in primary prostate cancer that are associated with lymph‐node invasion
Source: Clin Transl Med. 2025 Apr 11;15(4):e70288. doi: 10.1002/ctm2.70288 (PMC11992358; doi:10.1002/ctm2.70288)
Supplement: Supplementary file 1 — Supporting information [file CTM2-15-e70288-s001.docx]

**Transcriptomic miRNA and mRNA signatures in primary prostate cancer that are associated with lymph-node invasion**

Matias A. Bustos^1*^, Kelly K. Chong^1*^, Yoko Koh^1,2^, SooMin Kim^1,3^, Eleanor Ziarnik^3^, Romela I. Ramos^1^, Gianna Jimenez^2^, David L. Krasne^4^, Allen M. Warren^4^, Timothy G. Wilson^2^, and Dave S. B. Hoon^1,3^

^1^Department of Translational Molecular Medicine, Saint John’s Cancer Institute (SJCI) at Providence Saint John’s Health Center (SJHC), Santa Monica, California, USA

^2^Department of Urology and Urologic Oncology, SJCI at Providence SJHC, Santa Monica, California, USA

^3^Department of Genome Sequencing Center, SJCI at Providence SJHC, Santa Monica, California, USA

^4^Division of Surgical Pathology, Providence SJHC, Santa Monica, California, USA

*These authors equally contributed to this article.

**Correspondence:** Dave S. B. Hoon, Department of Translational Molecular Medicine, Saint John’s Cancer Institute (SJCI) at Providence Saint John’s Health Center (SJHC), Santa Monica, CA, USA.. [dave.hoon@providence.org](mailto:dave.hoon@providence.org)

**Funding**

This research program was supported by the generous donations of Mr. Jerome M. Applebaum and the Charles B. Fiscus Foundation as well as Ensign Cancer Research Foundation to Dept. of Urology and Urologic Oncology at Saint John’s Cancer Institute under TGW. We also thank the Henry L. Guenther Foundation’s generous support to the Dept. of Genomic Sequencing Center at Saint John’s Cancer Institute under DSBH.

ACKNOWLEDGEMENTS

The authors would thank to the staff of the Department of Translational Molecular Medicine and Genome Sequencing Center at SJCI for their kind advisory and technical assistance. This research program was supported by the generous donations of Mr. Jerome M. Applebaum and the Charles B. Fiscus Foundation as well as Ensign Cancer Research Foundation to Department of Urology and Urologic Oncology at Saint John’s Cancer Institute under TGW. We also thank the Henry L. Guenther Foundation’s generous support to the Dept. of Genomic Sequencing Center at Saint John’s Cancer Institute under DSBH.

AUTHOR CONTRIBUTIONS

Matias A. Bustos designed the study, performed analysis, organized and designed the figures, project management, and wrote the original manuscript. Kelly K. Chong performed bioinformatic and statistical analyses and wrote the original manuscript. Yoko Koh collected and prepared samples, conducted HTP and WTA assays, tissue pre-processing and microdissection, and wrote the original manuscript. SooMin Kim and Eleanor Ziarnik performed mRNA (HTP) and miR (WTA) library processing and sequencing. Romela I. Ramos project organization. Gianna Jimenez performed clinical data organization. David L. Krasne and Warren M. Allen performed pathological FFPE tissue samples review and data curation. Timothy G. Wilson clinical samples, clinical data organization, project management, clinical data review, and funding acquisition. Dave S. B. Hoon project organization and management. All authors reviewed, edited, and approved the final version of the manuscript

**Supplementary Information**

- 1. PCa tissue sample analyses

H&E-stained sections were reviewed by two experienced surgical pathologists in urology who marked the tumor area of PCa samples. H&E-stained sections were scanned using the Leica Application Suite X (version 3.7.4) and the areas of interest were measured using ImageJ (version 1.53t). For miR WTA and mRNA HTP assays, the FFPE sections were macrodissected using sterile scalpels to collect the regions of interest. If the area of the regions of interest was less than 22 mm^2^, additional FFPE sections were microdissected as required.

- 1. Dual application of WTA and HTP assays: tissue processing

The miR WTA and mRNA HTP were performed according to the respective HTG user manuals (Tuscon, AR). An appropriate volume of lysis buffer A containing proteinase K (PK) was added to 5 µm FFPE sections based on the tumor tissue area (mm^2^) measurements. The samples were then incubated at 56°C for 20 min at 850 rpm in a thermomixer to activate PK, followed by an additional incubation at 95°C for 15 min. Once incubation was completed, the samples were placed on ice for 10 min. Subsequently, 48 µL of lysed sample in a clean tube was mixed with DNase buffer and DNase enzyme and incubated at 37°C for 30 min. Then, 48 µL of biofluid lysis buffer was added and mixed by pipetting. The samples were incubated for 10 min at 95°C and then cooled on ice for 10 min. The prepared FFPE lysates were subsequently processed for both mRNA and miR whole transcriptomic analysis using the HTP assay reagent kit or the WTA assay reagent kit, respectively.

- 1. Dual application of WTA and HTP assays: library quality check

For both miR and mRNA analysis, the nucleic acids captured by the probes were tagged with unique indexes and underwent a PCR amplification process as previously described ^1^. All libraries were quantitated using the KAPA Library Quant Kit (Illumina Inc., San Diego, CA, USA) and the Universal qPCR Mix Kit (Roche, Basel, Switzerland) according to the manufacturer’s recommendations. Following a bead clean-up step, library size was quantified using a Qubit 4.0 fluorometer and quality control (QC) using the Agilent TapeStation 4200 with the High Sensitivity D1000 ScreenTape system. The expected peak size was between 150 and 170 base pairs. Samples that did not indicate proper library formation were excluded from sequencing. In all the runs, human brain total RNA (Ambion, Inc., Austin, TX, USA) was used as a control for library preparation, but the samples were not sequenced.

- 1. Dual application of WTA and HTP assays: NGS profiling of the libraries and quality control of NGS data

For each sequencing run, the samples were pooled and sequenced using Illumina MiSeq Reagent kit V3 for miR WTA analysis ^1^, and Illumina NextSeq550 High Output Kit v2.5 for mRNA HTP analysis, both with specifications for single-read sequencing of 50 cycles. FASTQ files were generated from raw sequencing data using Illumina BaseSpace BCL to FASTQ software version 2.2.0 and Illumina Local Run Manager Software. The Fastq files were parsed using HTG EdgeSeq HTG EdgeSeq Parser software version v5.1.724.4793 to generate the raw counts for 2,083 miRs per sample.

The NGS data for all the samples was analyzed for three QCs (QC0-2) using the HTG Reveal Software. The QC0 analyzed the RNA amount and quality. The QC1 determined the QC of the total reads per sample; and the QC2 displayed the biological variation among probe expression within a sample. All the samples passed the QC0-2 before being included in the analysis, except for PP89 for miR WTA dataset. The raw counts obtained for all the samples using the HTP and WTA assays were analyzed using principal components analysis (Figure S1A, H). The transcriptome mRNA and miR data generated using the HTP and WTA assays are provided within the supporting information files (**Tables S13-15**). One patient (PP89) failed QC for miR WTA dataset, and it was removed from the analysis (**Tables S14**).

**Supplementary Figures**


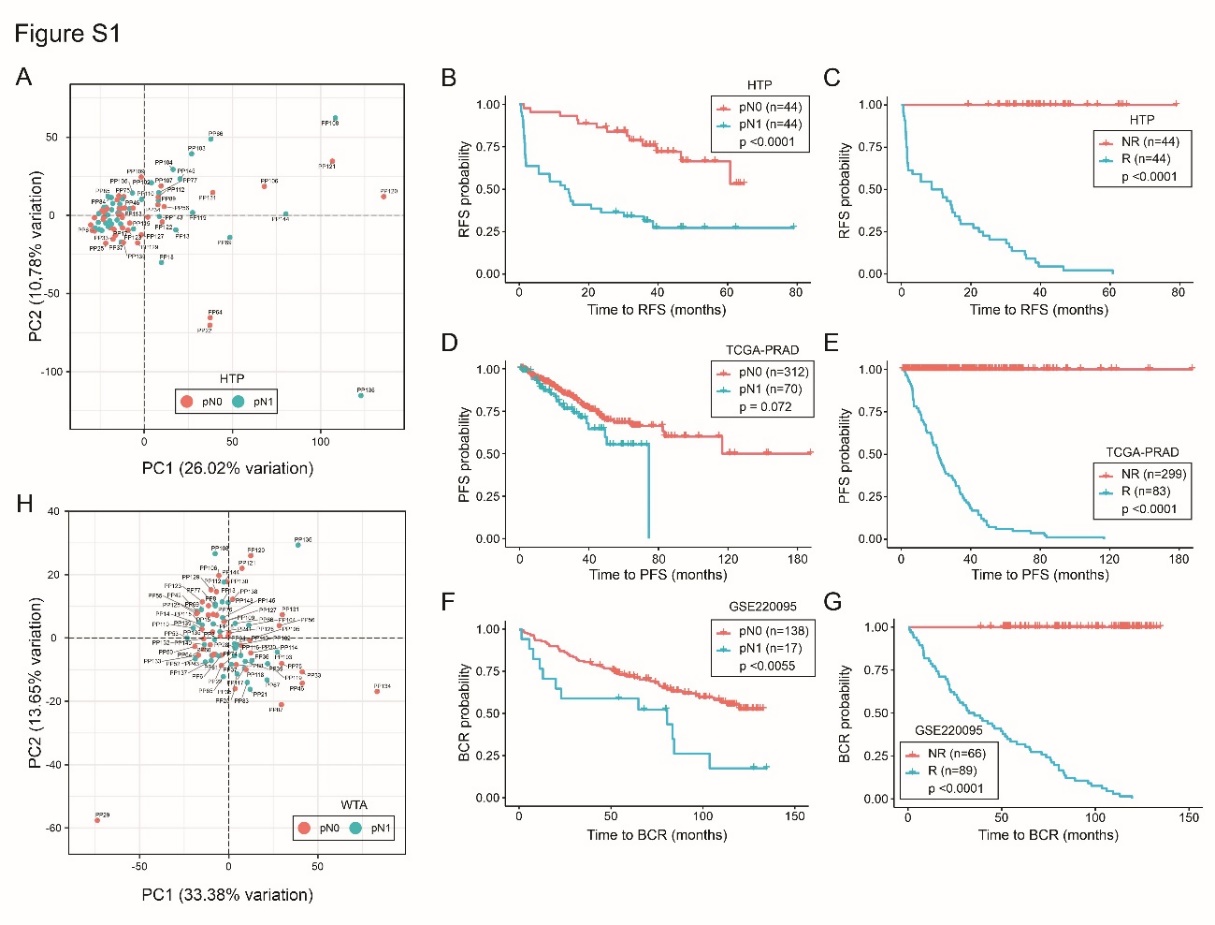


**Fig. S1.** Clinical outcomes of the cohorts utilized in the study. (A) Principal component analysis (PCA) showing the distribution of the PCa tissue samples analyzed by HTP dataset. (B) Kaplan-Meier curves showing the relapse-free survival (RFS) probability comparing patients in pN0 vs. pN1 groups. (C) Kaplan-Meier curves showing the RFS probability comparing patients in recurrent (R) vs. non-recurrent (NR) groups. (D) Kaplan-Meier curves showing the progression-free survival (PFS) probability comparing patients in pN0 vs. pN1 groups from the TCGA-PRAD database. (E) Kaplan-Meier curves showing the PFS probability comparing patients in R vs. NR groups from the TCGA-PRAD database. (F) Kaplan-Meier curves showing the biochemical recurrence (BCR) probability comparing patients in pN0 vs. pN1 groups from the GSE220095 dataset. (G) Kaplan-Meier curves showing the BCR probability comparing patients in R vs. NR groups from the GSE220095 dataset. (H) PCA showing the distribution of the PCa tissue samples analyzed by WTA dataset. Statistical analysis: Log-rank test for a-f.


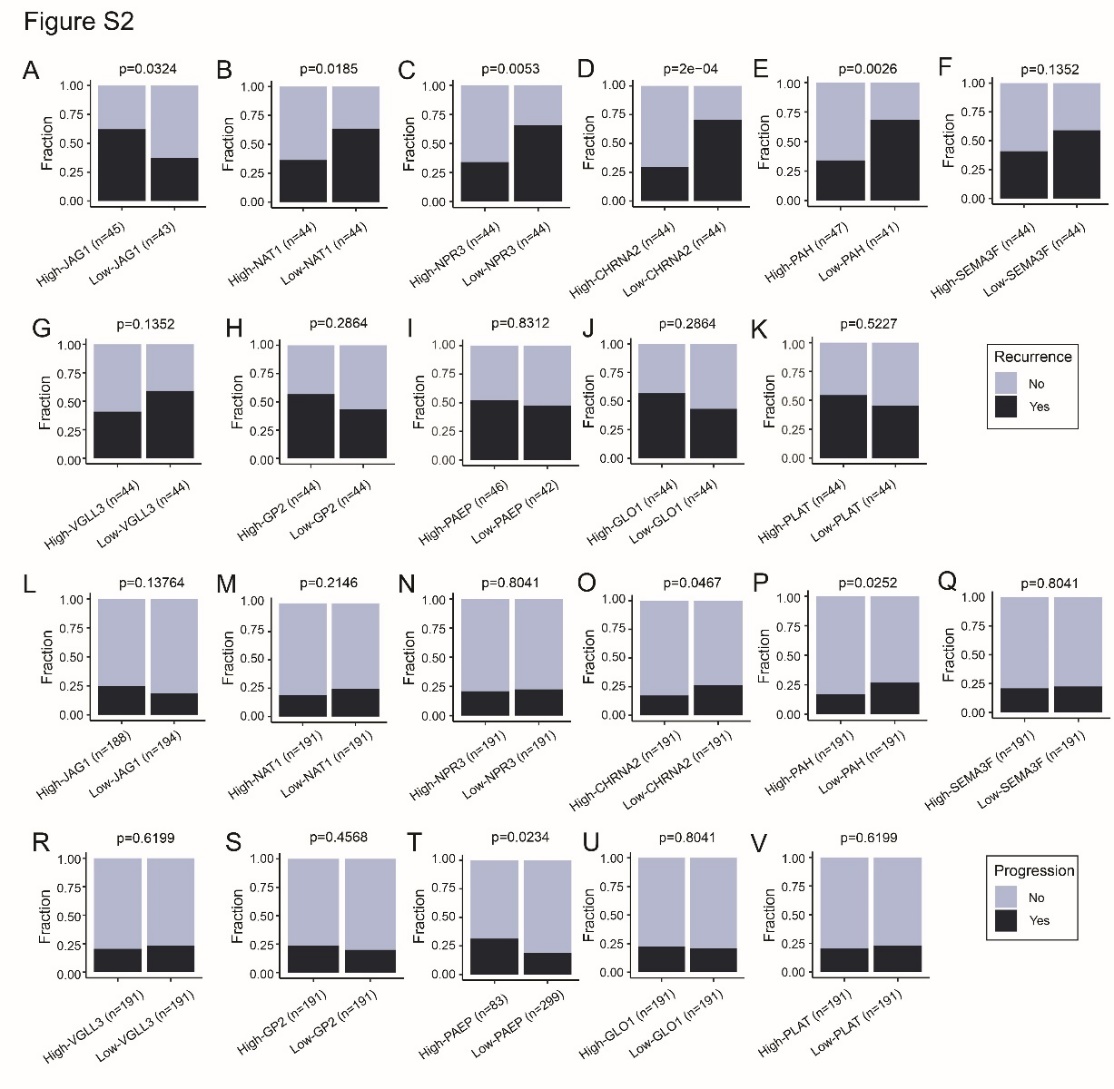


**Fig. S2.** Gene expression levels and association with pN1 status in the HTP and TCGA-PRAD datasets. (A-K) Stacked bar plots showing the distribution of patients with or without recurrence in low-*JAG1* vs. high-*JAG1* (A); low-*NAT1* vs. high-*NAT1* (B); low-*NPR3* vs. high-*NPR3* (C); low-*CHRNA2* vs. high-*CHRNA2* (D); low-*PAH* vs. high-*PAH* (**e**); low-*SEMA3F* vs. high-*SEMA3F* (F); low-*VGLL3* vs. high-*VGLL3* (G); low-*GP2* vs. high-*GP2* (H); low-*PAEP* vs. high-*PAEP* (I); low-*GLO1* vs. high-*GLO1* (J); low-*PLAT* vs. high-*PLAT* (K); groups. (L-V) Progression status between patients with low-*JAG1* vs. high-*JAG1* (L); low-*NAT1* vs. high-*NAT1* (M); low-*NPR3* vs. high-*NPR3* (N); low-*CHRNA2* vs. high-*CHRNA2* (O); low-*PAH* vs. high-*PAH* (P); low-*SEMA3F* vs. high-*SEMA3F* (Q); low-*VGLL3* vs. high-*VGLL3* (R); low-*GP2* vs. high-*GP2* (S); low-*PAEP* vs. high-*PAEP* (T); low-*GLO1* vs. high-*GLO1* (U); low-*PLAT* vs. high-*PLAT* (V). Statistical analysis: Fisher’s exact test for A-V.


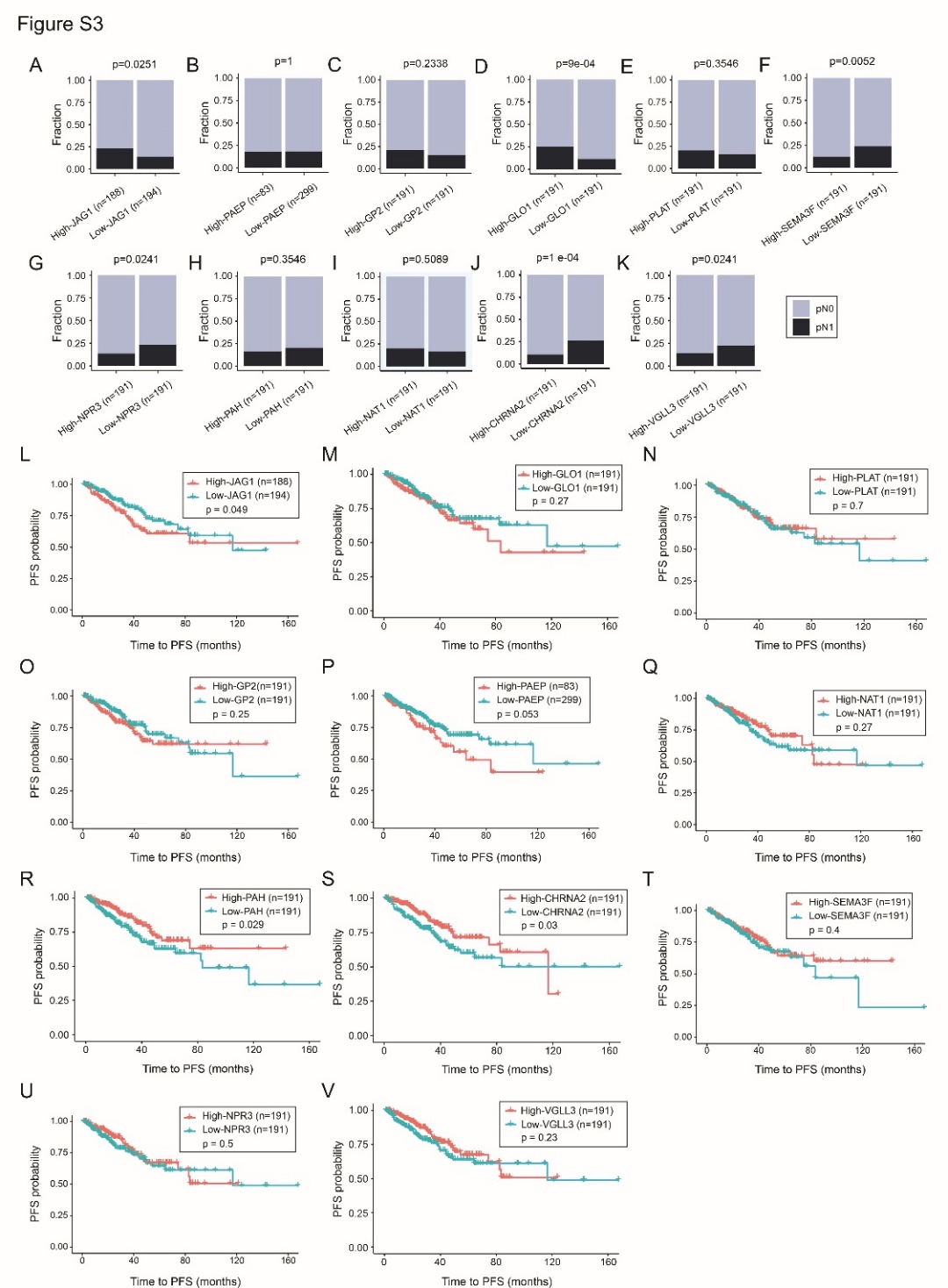


**Fig. S3.** Association between the 11 DEGs found in pN1 with LNI status and progression-free survival (PFS) in the TCGA-PRAD dataset. (A-K) Stacked bar plots showing the distribution of pN1 and pN0 patients with low- vs high- levels considering *JAG1* (A); *PAEP* (B); *GP2* (C) *GLO1* (D); *PLAT* (E); *SEMA3F* (F); *NPR3* (G); *PAH* (H); *NAT1* (I); *CHRNA2* (J); *VGLL3* (K) groups. (L-V). Kaplan-Meier curves showing the PFS probability comparing patients with low- vs high- levels considering *JAG1* (L); *GLO1* (M); *PLAT* (N); *GP2* (O); *PAEP* (P); *NAT1* (Q); *PAH* (R); *CHRNA2* (S); *SEMA3F* (T); *NPR3* (U); and *VGLL3* (**v**) using the HTP dataset. Statistical analysis: Fisher’s exact test for A-K and Log-rank test for L-V.


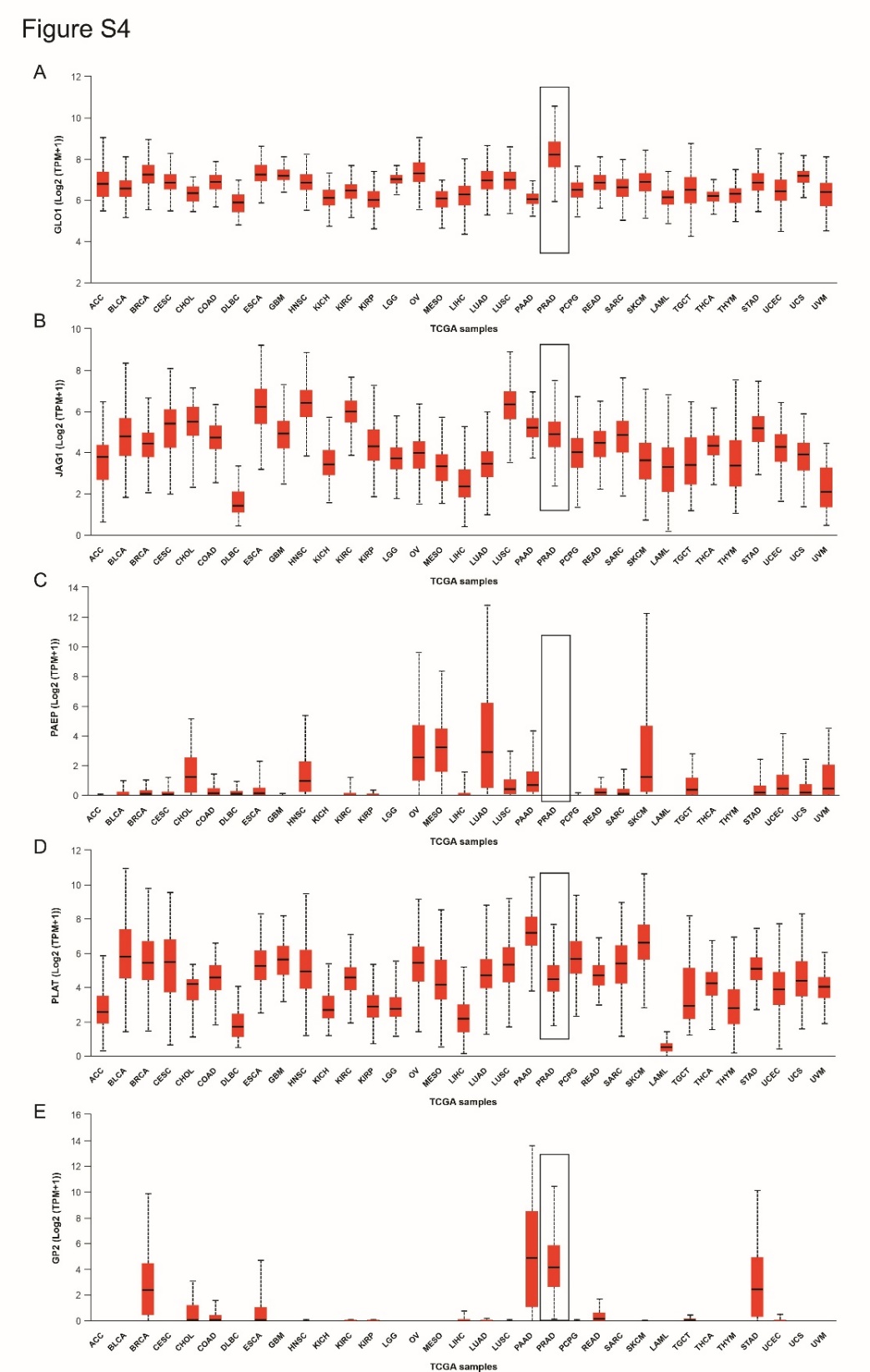


**Fig. S4.** The mRNA levels of the five upregulated genes in the 33 tumor types of the TCGA database. (A-E) The mRNA levels (Log2 TPM+1) of *GLO1* (A), *JAG1* (B), P*AEP* (C), *PLAT* (D), *GP2* (E) in the 33 tumor types of the TCGA database.


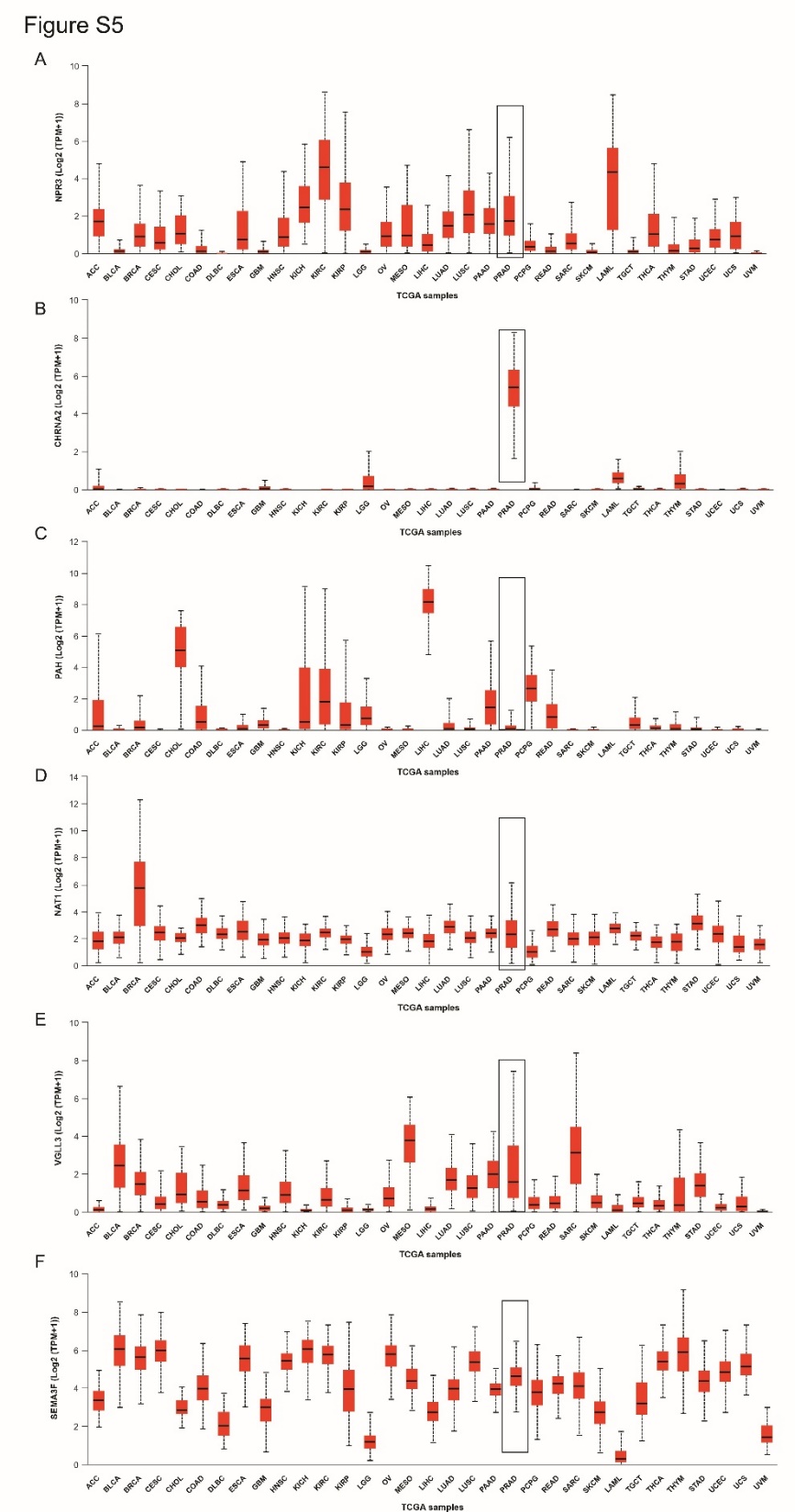


**Fig. S5.** The mRNA levels of the six downregulated genes in the 33 tumor types of the TCGA database. (A-F) The mRNA levels (Log2 TPM+1) of *NPR3* (A), *CHRNA2* (B), *PAH* (C), *NAT1* (D), *VGLL3* (E), *SEMA3F* (F) in the 33 tumor types of the TCGA database.


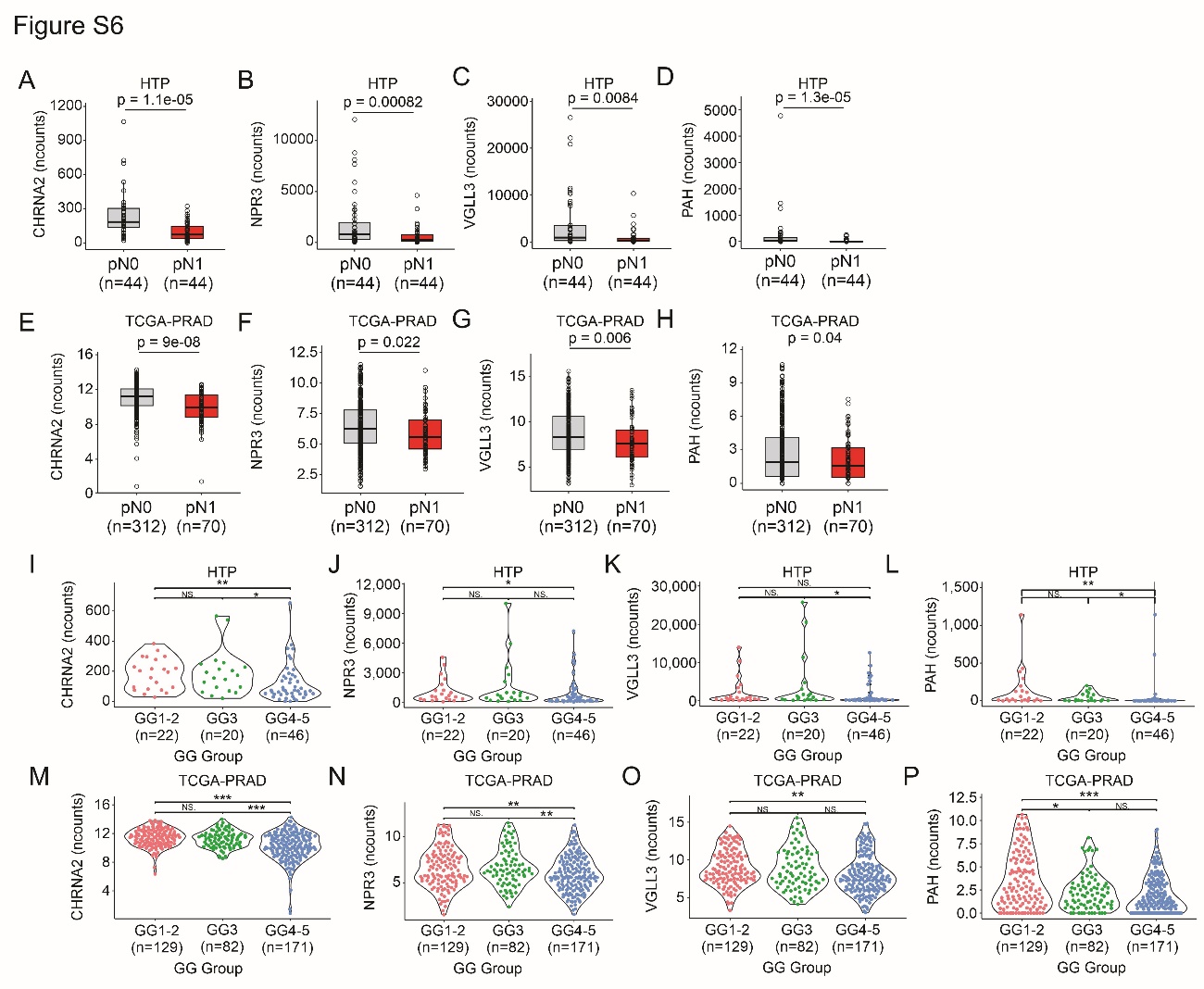


**Fig. S6.** Expression levels of the four DEGs consistently found in PCa tissue samples. (A-D) Boxplots showing the mRNA levels of *CHRNA2* (A), *NPR3* (B), *VGLL3* (C), and *PAH* (D) in pN0 vs. pN1 groups in the HTP dataset. (E-H) Boxplots showing the mRNA levels of *CHRNA2* (E), *NPR3* (F), *VGLL3* (G), and *PAH* (H) in pN0 vs pN1 groups in the TCGA-PRAD dataset. (I-L) Violin plots showing the mRNA levels for *CHRNA2* (I), *NPR3* (J), *VGLL3* (K), and *PAH* (L) in GG1-2, GG3, and GG4-5 groups in the HTP dataset. (M-L) Violin plots showing the mRNA levels for *CHRNA2* (M), *NPR3* (N), *VGLL3* (O), and *PAH* (P) in GG1-2, GG3, and GG4-5 groups in the TCGA-PRAD dataset. Statistical analysis: Wilcoxon t-test analysis for A-H. One way ANOVA test and Kruskal-Wallis post-hoc test for I-P. * *p* < 0.05; ** *p* < 0.01; *** *p* < 0.001.


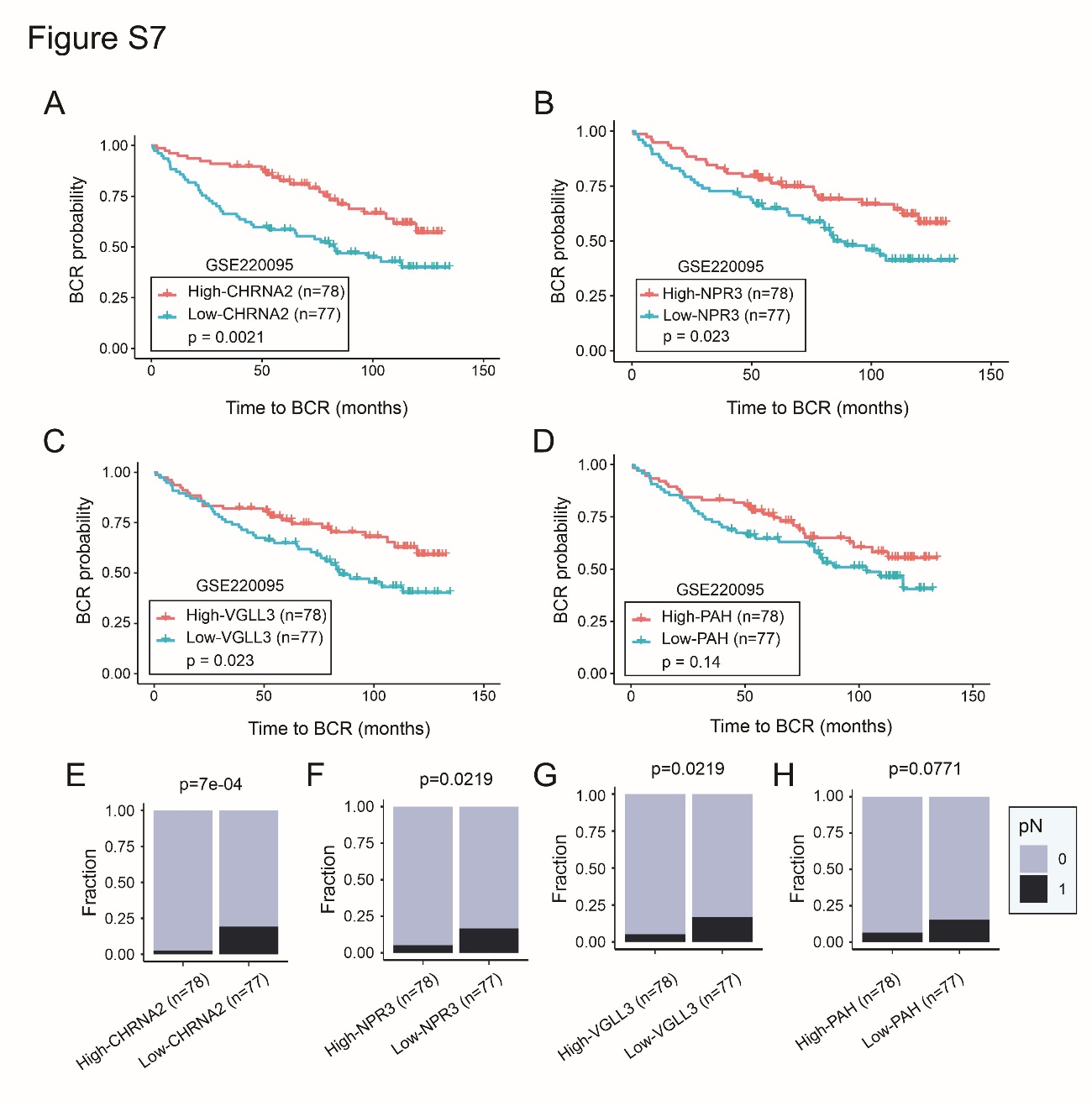


**Fig. S7.** Association between the four-gene signature and clinical outcomes. (A-D) Kaplan-Meier curves showing the biochemical recurrence (BCR) probability comparing patients with low- vs high- levels considering *CHRNA2* (A); *NPR3* (B); *VGLL3* (C); and *PAH* (D) using the GSE220095 dataset. (E-H) Stacked bar plots showing the distribution of pN1 and pN0 patients with low- vs high- levels considering *CHRNA2* (E); *NPR3* (F); *VGLL3* (G); and *PAH* (H) groups using the GSE220095 dataset. Statistical analysis: p-adjusted values for A-C. Fisher’s exact test for E-H and Log-rank test for A-D.

**
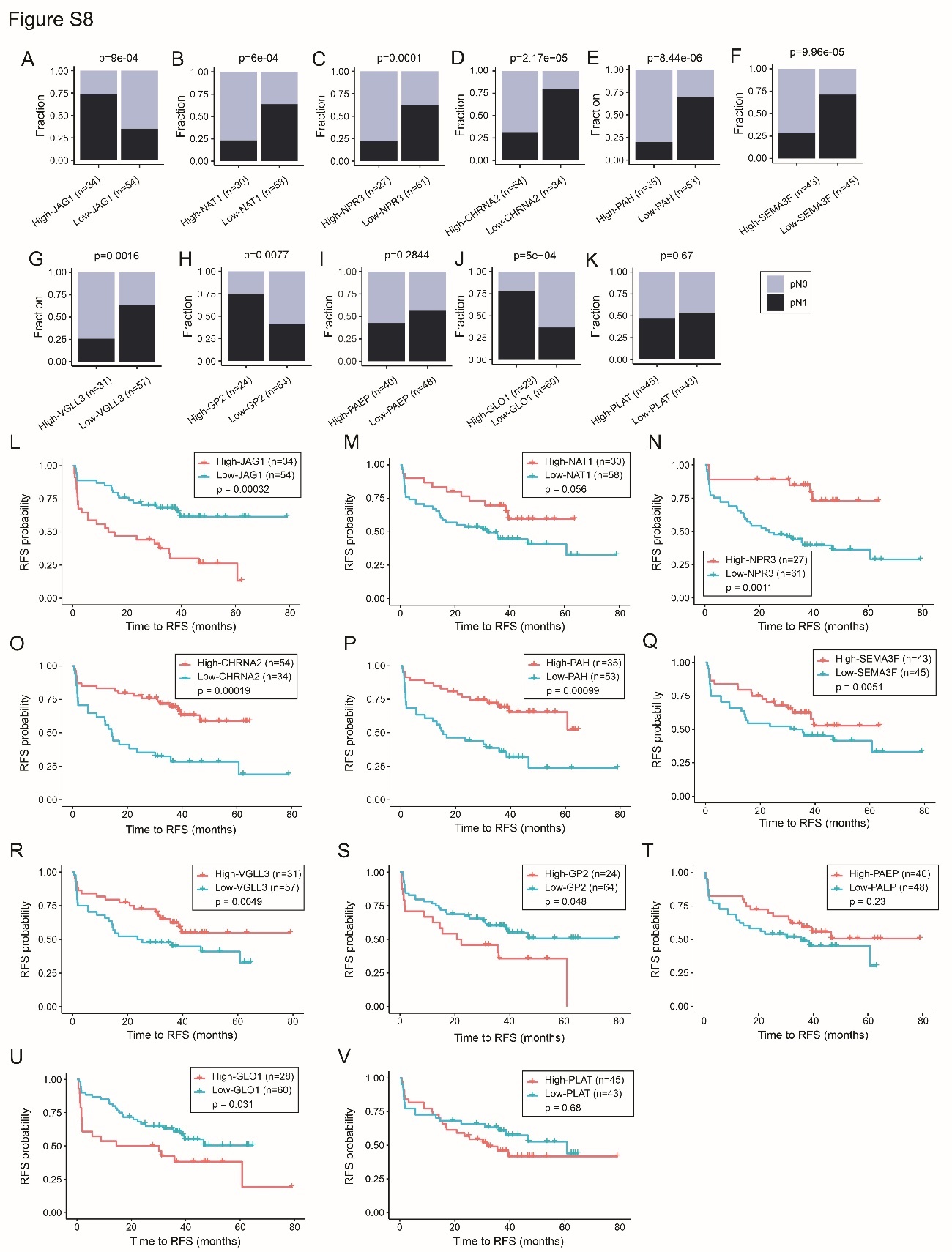
**

**Fig. S8.** Association between the DEGs found in pN1 and relapse-free survival (RFS). A-K Stacked bar plots show the distribution of pN1 and pN0 patients in low- vs high- levels considering *JAG1* (A); *NAT1* (B); *NPR3* (C); *CHRNA2* (D); *PAH* (E); *SEMA3F* (F); *VGLL3* (G); *GP2* (H); *PAEP* (I); *GLO1* (J); or *PLAT* (K) genes expression based on ROC best values cutoff using the HTP dataset. L-V Kaplan-Meier curves showing the RFS probability comparing patients with low- vs high- levels considering *JAG1* (L); *NAT1* (M); *NPR3* (N); *CHRNA2* (O); *PAH* (P); *SEMA3F* (Q); *VGLL3* (R); *GP2* (S); *PAEP* (T); *GLO1* (U); or *PLAT* (V) genes expression based on ROC best values cutoff using the HTP dataset.


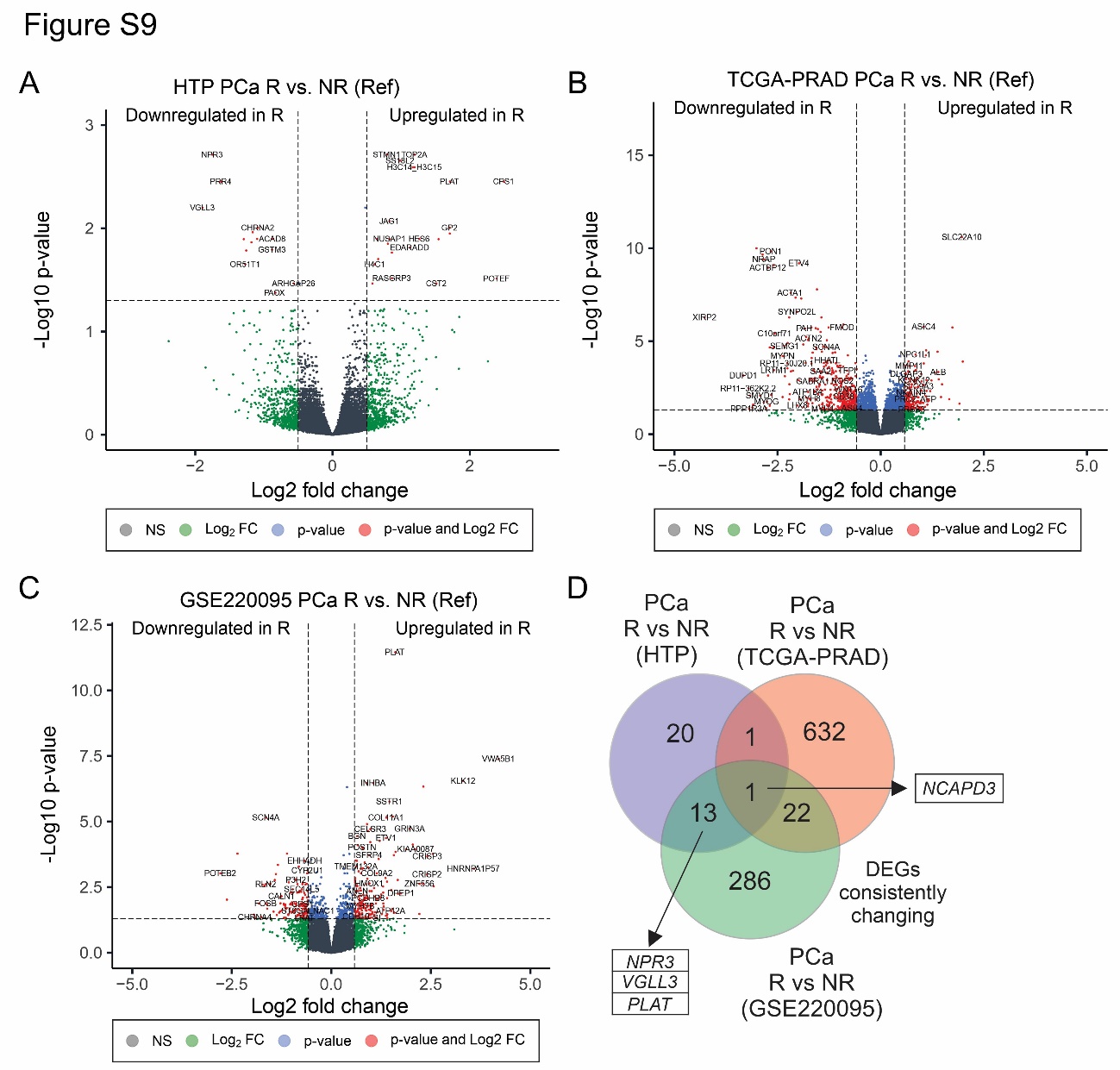


**Fig. S9.** Common DEGs in PCa biopsies and tumor tissues from recurrent and non-recurrent primary PCa tumors. (A-C) Volcano plots showing the differential expressed genes (DEGs) between (A) primary PCa tumors from recurrent (R, n = 44) vs. non-recurrent (NR, n = 44) patients from the HTP dataset; (B) primary PCa tumors from R (n = 70) vs. NR (n = 312) patients from the TCGA-PRAD dataset; and (C) tissue biopsies from primary PCa tumors from recurrent (n = 66) vs. non-recurrent (n = 89) patients from the GSE220095 dataset. (D) Overlapping DEGs in R vs. NR using the HTP, TCGA-PRAD, and GSE220095 datasets.


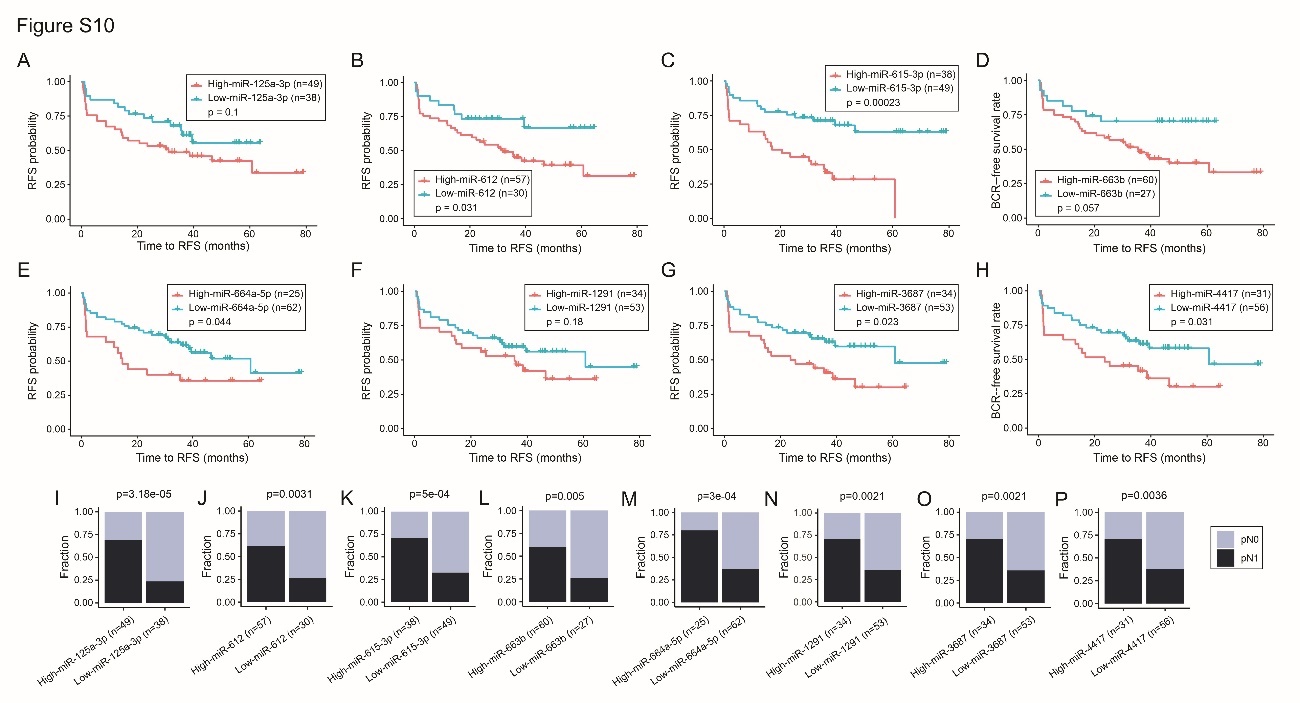


**Figure S10.** Upregulated miRs found in primary PCa tumors that developed LNI were associated with RFS and pN status. (A-H) Kaplan-Meier curves showing the relapse-free survival (RFS) probability comparing patients with low- vs high- levels of miR-125a-3p (A); miR-612 (B); miR-615-3p (**c**); miR-663b (D); miR-664a-5p (E); miR-1291 (F); miR-3687 (G); miR-4417 (H) expression based on ROC best values cutoff in the HTP dataset. (I-P) Stacked bar plots showing the distribution of pN1 and pN0 patients with low- vs high- levels of miR-125a-3p (I); miR-612 (J); miR-615-3p (K); miR-663b (L); miR-664a-5p (M); miR-1291 (N); miR-3687 (O); and miR-4417 (P) expression based on ROC best values expression cutoff in the HTP dataset.


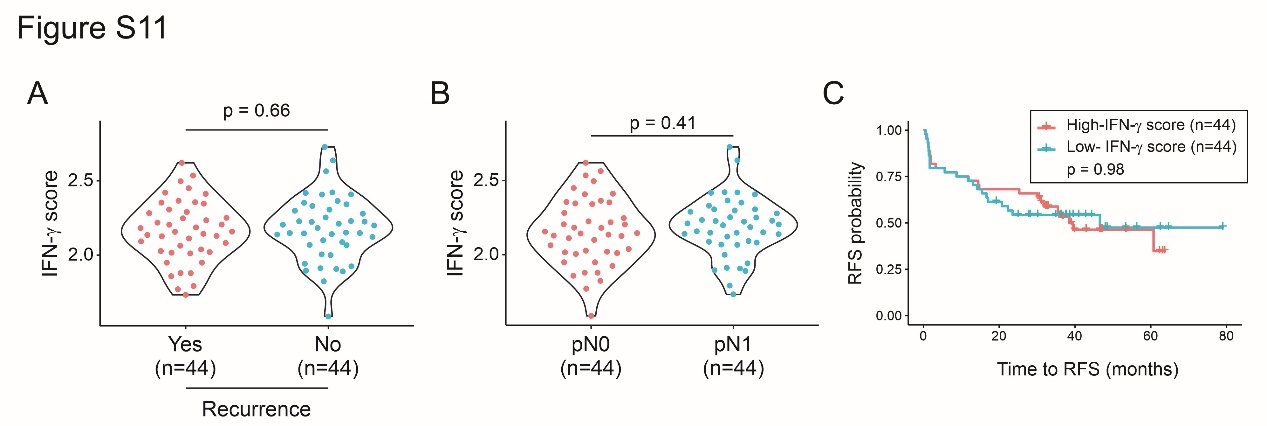


**Fig. S11.** Analysis of the IFN-γ score and recurrence status, pN status, or RFS. (A) IFN-γ score in PCa patients who were recurrent vs. non recurrent. (B) IFN-γ scores levels in pN1 vs. pN0 PCa patients. (C) Kaplan-Meier curve comparing PCa patients that were divided based on high-IFN-γ score or low-IFN-γ score. Statistical analysis: Log-rank test for **c** and Wilcoxon U test for A-C.


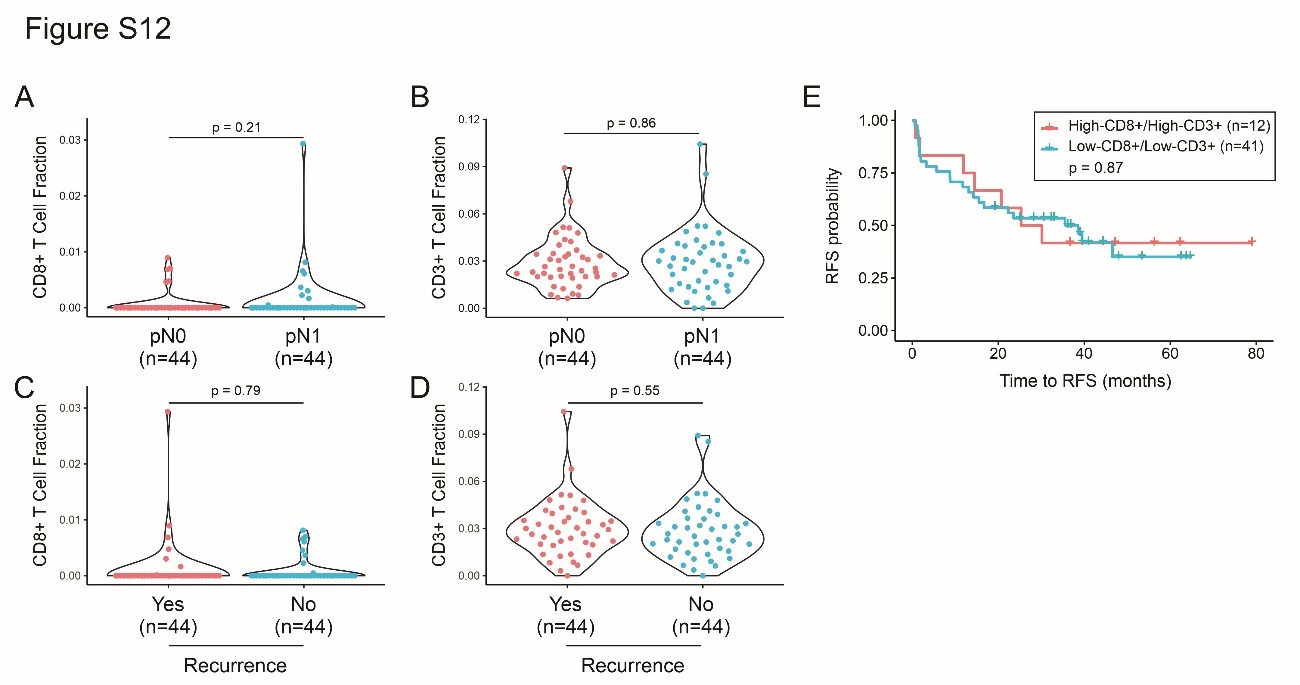


**Fig. S12.** Correlation between CD8^+^ or CD3^+^ T cells and pN status, recurrence status, and RFS. (A-B) Fractions of CD8^+^ (A) or CD3^+^ (B) T cells in pN1 vs. pN0 groups. (C-D) Fraction of CD8^+^ (C) or CD3^+^ (D) T cells in patients who recurred vs. patients that did not recur. (E) Kaplan-Meier curve comparing PCa patients that were divided based on high-CD8^+^/high-CD3^+^ vs. low-CD8^+^/low-CD3^+^. Statistical analysis: Log-rank test for **e**, and Wilcoxon U test for A-D.

**
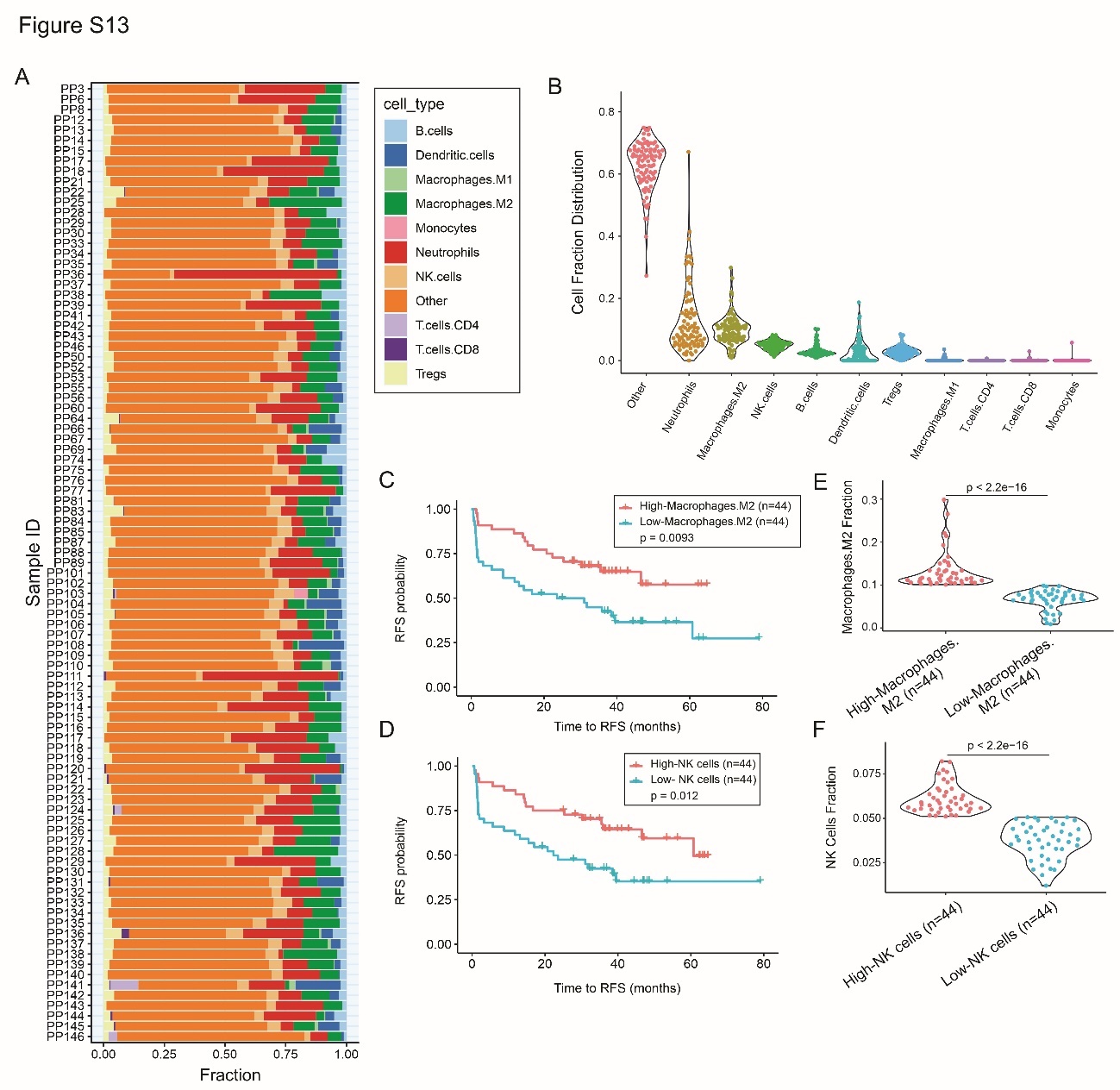
**

**Fig. S13.** Immune cell types found in primary PCa tumor that were associated with clinical outcomes. (A-B) Immune cell fractions for each primary PCa tumor analyzed. (C-D) Kaplan-Meier curves comparing RFS probability of PCa patients that were divided based on low- vs high- levels of Macrophages.M2 (C) or NK cells (D). (E-F) Violin plots showing the Macrophages.M2 (E) and NK cells (F) levels in each group analyzed.


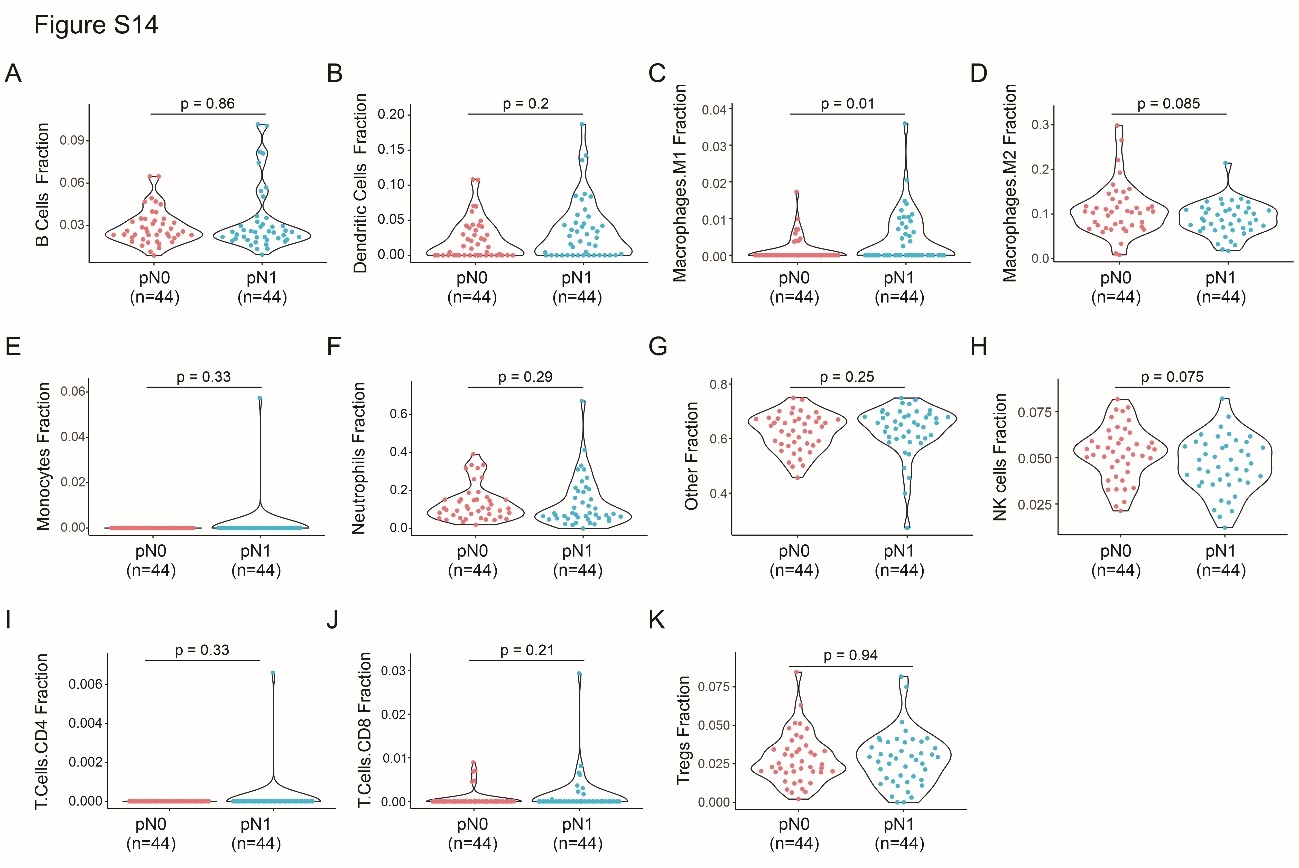


**Fig. S14.** Immune cell fractions of primary PCa tumors using quanTIseq algorithm. (A-K) Immune cell fractions using CODEFACs deconvolution analysis for B cells (A), Dendritic cells (B), Macrophages.M1 (C), Macrophages.M2 (D), Monocytes (E), Neutrophils (F), Other (uncharacterized cells) (G), NK cells (H), T cells CD4^+^ (I), T cells CD8^+^ (J), Tregs (K), in primary tumors of PCa patients that were divided into pN0 and pN1. Statistical analysis: Wilcoxon U test for A-K.

**Supplementary Tables**

**Table S1.** DEGs in pN1 vs. pN0 groups using the HTP dataset.

**Table S2.** DEGs in pN1 vs. pN0 groups using the TCGA-PRAD dataset.

**Table S3.** DEGs in pN1 vs. pN0 groups using the GSE220095 dataset.

**Table S4.** Consistent DEGs in pN1 vs pN0 overlap in all the HTP, TCGA-PRAD, and GSE220095 datasets.

**Table S5.** Summary of the metric used to evaluate the models across the study.

**Table S6.** Summary of the ROC cutoff for the DEG and DE miRs identified in primary tumors diagnosed as pN1.

**Table S7.** DEGs in recurrent vs. non-recurrent groups from the HTP dataset.

**Table S8.** DEGs in recurrent vs. non-recurrent groups from the TCGA-PRAD dataset.

**Table S9.** DEGs in recurrent vs. non-recurrent groups from the GSE220095 dataset.

**Table S10.** DE miRs in pN1 vs. pN0 PCa tissue samples using the WTA dataset.

**Table S11.** The spearman correlation values between DE miRs and the DEGs in pN1 compared to pN0 groups in the respective WTA and HTP datasets.

**Table S12.** Correlation between DE miRs and DEGs in pN1 group using the HTP and WTA datasets.

**Table S13.** The DEGs found in pN1 are predictive targets of specific miRs of the WTA dataset.

**Table S14.** Summary of the tissue samples analyzed for mRNA and miR in the HTP and WTA datasets.

**Table S15.** Data for the tissue samples analyzed for miR using WTA dataset.

**Table S16.** Data for the tissue samples analyzed for mRNA using HTP dataset.

**References**

1. Tran KD, Gross R, Rahimzadeh N, Chenathukattil S, Hoon DSB, Bustos MA. Assessment of Cell-Free microRNA by NGS Whole-Transcriptome Analysis in Cutaneous Melanoma Patients' Blood. *Methods in molecular biology*. 2021;2265:475-486. doi:10.1007/978-1-0716-1205-7_34
